# Venom vesicles from the parasitoid *Ganaspis hookeri* facilitate venom protein entry into host immune cells

**DOI:** 10.1371/journal.ppat.1014707

**Published:** 2026-10-16

**Authors:** Nicholas M. Bretz, Chris Lark, Sarah Perkel, Rebecca Jackson, Jeremy D. Driskell, James A. Mobley, Nathan T. Mortimer

**Affiliations:** 1 Department of Biochemistry & Biophysics, Oregon State University, Corvallis, Oregon, United States of America; 2 School of Biological Sciences, Illinois State University, Normal, Illinois, United States of America; 3 Electron Microscopy Facility, Oregon State University, Corvallis, Oregon, United States of America; 4 Department of Chemistry, Illinois State University, Normal, Illinois, United States of America; 5 Department of Anesthesiology and Perioperative Medicine, University of Alabama at Birmingham School of Medicine, Birmingham, Alabama, United States of America; Medical College of Wisconsin, UNITED STATES OF AMERICA

## Abstract

The parasitoid wasp *Ganaspis hookeri* infects *Drosophila melanogaster* larvae, laying an egg and injecting venom directly into the host body cavity*.* While infected hosts mount an immune response in an attempt to eliminate the parasitoid egg, parasitoid venom proteins act to inhibit these host responses and manipulate host physiology to ensure infection success. A key immune suppressive venom protein in *G. hookeri* is a venom-specific isoform of the SERCA (Sarco/endoplasmic reticulum calcium-ATPase) calcium pump. However, SERCA is a large hydrophobic protein, and the mechanism by which it and other venom proteins are transported into host cells is not well understood. Electron microscopy and nanoparticle tracking analysis of *G. hookeri* venom revealed the presence of venom extracellular vesicles (vEVs) as a putative transport mechanism. We characterized the biophysical properties of vEVs, and found that *G. hookeri* venom contains multiple vesicle types that are distinguishable by size, zeta potential, protein content, and density. A subset of these vEVs contain SERCA, and we found that fluorescently labeled vEVs are able to enter into host immune cells and alter host intracellular calcium levels, and that disruption of vesicle integrity results in the loss of this SERCA activity in host cells. Together, our data support a model in which *G. hookeri* venom proteins, including SERCA, are packaged into an array of venom vesicles, and are transported into host cells for immune suppression.

## Author summary

Upon infection, parasites introduce virulence factors into the host cells in an attempt to overcome the host immune response. While many virulence proteins are easily secreted, large, hydrophobic virulence factors have been identified in many parasites, including in the venoms of parasitoid wasps. We used the *Drosophila melanogaster*-parasitoid wasp experimental system to identify and characterize the packaging of venom proteins into venom extracellular vesicles in the parasitoid *Ganaspis hookeri.* We further found that disruption of vesicle integrity inhibits the anti-immune function of venom. The use of extracellular vesicles to transport virulence proteins is widely conserved, including among human-infecting parasites, and our biophysical characterization of venom extracellular vesicles opens the door for understanding this understudied aspect of infection biology.

## Introduction

Parasitoid wasps comprise an ecologically diverse group of insects numbering an estimated 350,000 species worldwide [1]. These parasitoids infect a wide range of insect host species, and while virulence strategies differ dependent on the host and parasitoid species, wasps use an array of virulence factors, including venom proteins, calyx proteins, and symbiont polydnaviruses to overcome host defense responses and ensure offspring fitness [2–4]. These parasitoid virulence factors target host immunity using a wide range of mechanisms including the destruction of immune cells or immune cell precursors, dysregulation of the immune cell actin cytoskeletal structure, as well as altering the adhesion of immune cells [5–9]. Parasitoid venoms also enhance offspring fitness through manipulation of host development and metabolism [10–13].

The parasitoid *Ganaspis hookeri* is a generalist of *Drosophila* species, with high infection success against *Drosophila melanogaster* [14]. *G. hookeri* is an endoparasitoid of *Drosophila* larvae, and during infection, lays an egg and injects virulence factor containing venom into the host hemocoel. Following parasitoid infection, *D. melanogaster* larvae mount a conserved cellular immune response against the wasp egg [15,16]. This response is initiated by an increase in intracellular calcium levels in host plasmatocytes, a class of circulating hemocytes with immune functions similar to mammalian macrophages [14,17]. This ‘calcium burst’ results in calcium-signaling mediated immune activation enabling plasmatocytes to migrate towards and bind to the wasp egg, forming an inner capsule that is later encapsulated by lamellocytes, a parasitoid induced hemocyte class, and melanized leading to parasitoid death [18–20].

*G. hookeri* venom consists of a complex mix of proteins with a wide range of predicted functions [14,21]. We have shown that *G. hookeri* infection inhibits host plasmatocyte activation utilizing a venom-specific isoform of SERCA (Sarco/endoplasmic reticulum calcium-ATPase) [14]. SERCA is widely conserved, and acts as an active calcium pump, removing calcium ions from the cytoplasm into SR/ER stores [22]. Our previous work showed that *G. hookeri* infection antagonizes the plasmatocyte calcium burst, and that this inhibition is dependent on venom SERCA activity, suggesting that venom SERCA functions in host plasmatocytes to lower cytoplasmic calcium levels [14]. Venom SERCA is a 110 kDa protein with 11 predicted transmembrane domains, which leads to an interesting problem: our results suggest that venom SERCA, despite being a large, hydrophobic protein, is transported through aqueous environments of the venom and host hemocoel before it enters host cells to manipulate calcium signaling.

A wide range of parasite species, including other species of *Drosophila-*infecting parasitoid wasps, appear to have solved this problem through the evolution of virulence factor containing extracellular vesicles (EVs). EVs are broadly used by parasites to transfer virulence factors to host cells, where they modulate host immunity and physiology [23–25]. In the case of parasitoid wasps, these EVs are found within the venom and are known as venom extracellular vesicles (vEVs) [9,23,26,27]. *Leptopilina heterotoma*, a generalist parasitoid of *Drosophila* species, utilizes vEVs, also referred to as virus-like particles, to infect *Drosophila* hosts. *L. heterotoma* vEVs are largely homogenous, with an estimated size of 300 nm and displaying a knob-like morphology [23,28,29]. These *L. heterotoma* vEVs interact with host hemocytes to induce lamellocyte lysis and cell death within the hematopoietic lymph gland [5,9]. Similarly, the venom of *Leptopilina boulardi*, a specialist parasitoid of melanogaster subgroup larvae, also contains vEVs (more commonly referred to as venosomes) [26,27]. *L. boulardi* vEVs are less well characterized, but exhibit sizes ranging from 100 nm - 200 nm as measured by electron microscopy [27]. *L. boulardi* employs a RhoGAP domain protein that is predicted to disrupt actin cytoskeletal architecture and adhesion properties of host hemocytes, and this protein is found within *L. boulardi* vEVs [27,30]. Based on the widespread use of EV-mediated parasitoid virulence factor delivery, we hypothesize that *G. hookeri* venom contains vEVs to enable the transport of venom SERCA and associated venom proteins into host hemocytes during infection. To test our hypothesis, we utilized a combination of biochemical, biophysical, and cell biological assays to characterize the contents of *G. hookeri* venom.

## Results

### SERCA is expressed in *G. hookeri* venom gland cells

To determine the localization of SERCA expression in *G. hookeri* venom producing cells, whole venom apparatuses from female *G. hookeri* wasps were dissected, stained with anti-SERCA and fluorescently-labeled phalloidin, and imaged using confocal microscopy (Fig 1A-C; bright field S1 Fig). Maximum intensity projections of Z-stack images taken at 40x magnification reveal that SERCA is expressed in cells of the venom gland (Fig 1A; indicated by the arrow), and is also found within the venom reservoir (Fig 1A; indicated by the arrowhead). SERCA localization appears to be non-random within the venom gland, but rather shows a distinct localization pattern throughout the gland. To better characterize this SERCA localization, stained venom glands were imaged at 63x magnification. Z-stack images were projected through three dimensions, and 2D slices of the projection were analyzed for SERCA localization (Fig 1D-F). The projection shows that the SERCA stain appears to associate with actin structures within the venom gland (Fig 1D-F; indicated by arrow). The *L. heterotoma* venom gland is organized around actin channels which transport venom proteins for secretion [31]. This organization allows *L. heterotoma* venom proteins to be packaged into vEVs, and we hypothesize that venom SERCA might be similarly packaged in *G. hookeri* venom glands.

**Fig 1:**
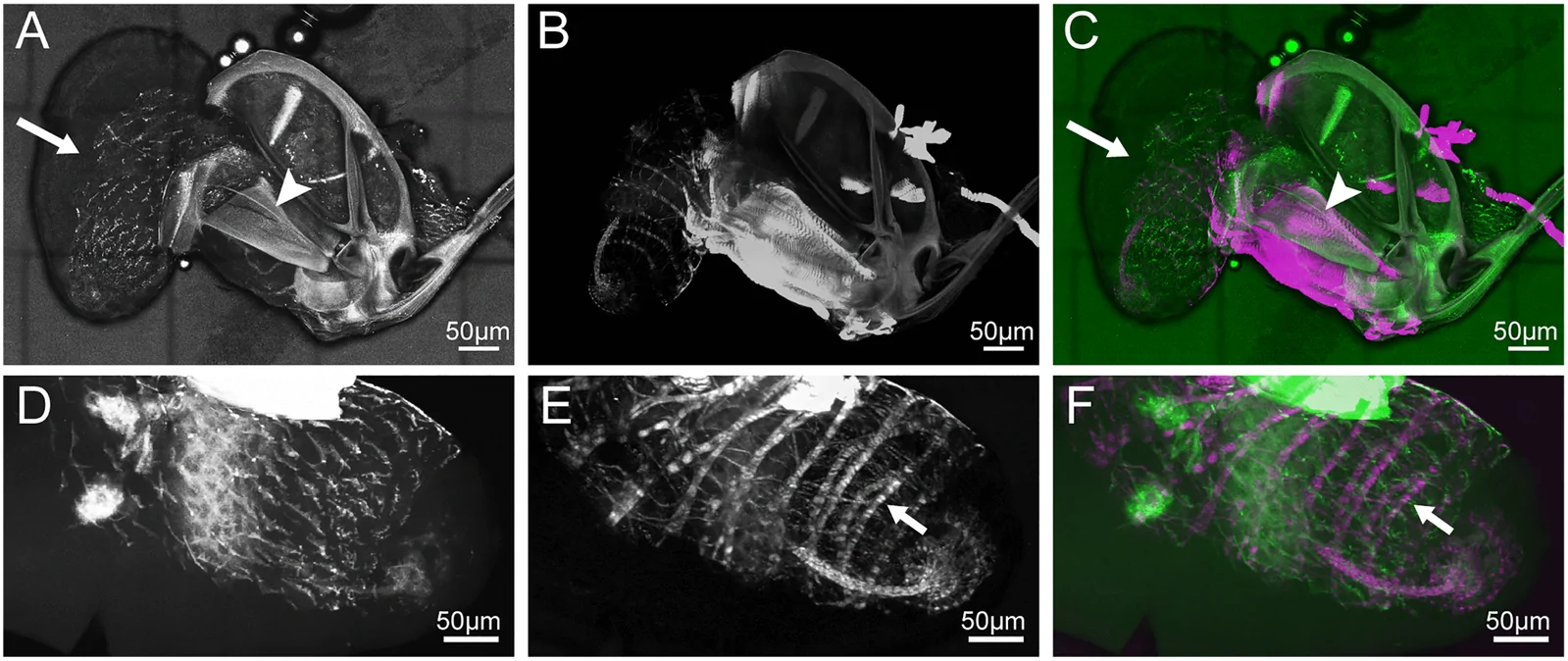
Confocal micrographs of SERCA staining in *G. hookeri* venom apparatuses. **(A-C)** Maximum projection of Z-stack images taken at 40x magnification. (A) anti-SERCA, (B) phalloidin-Alexa Fluor 633, and (C) merged. Arrows in A, C indicate the venom gland and arrowheads in A, C indicate the venom reservoir. **(D-F)** 3D projection of Z-stack images of the venom gland taken at 63x magnification. (D) anti-SERCA, (E) phalloidin-Alexa Fluor 633, and (F) merged. Arrows in E-F indicate actin structures. Scale bars are 50 µm.

### Characterization of *G. hookeri* venom reveals a heterogenous population of vEVs

To determine whether *G. hookeri* venom contains vesicles, purified whole venom was characterized with scanning electron microscopy (SEM; Fig 2A) and negative stain transmission electron microscopy (TEM; Fig 2B). We observed a variety of extracellular particles in venom, with a range of sizes and morphologies including putative extracellular vesicles (EVs) and non-vesicular extracellular particles [32]. Many of the particles are < 40 nm, and are unlikely to be membrane enclosed vesicles (Fig 2A, example indicated by the arrow). We also observed a range of particles with sizes between 40–200 nm that appear to correspond to EVs (Fig 2A, examples indicated by arrowheads), as well as aggregates of these EV-sized particles (S2A-B Fig). Based on size, these *G. hookeri* vesicles can best be characterized as small EVs (< 200 nm) [32]. These vesicles are considerably smaller than the vEVs described in *L. heterotoma*, and appear polydisperse, in contrast to the uniform size distribution observed for vEVs in both *L. heterotoma* and *L. boulardi*. These observations further support the conclusion that *G. hookeri* venom contains vesicles, and suggests that *G. hookeri* venom contains a heterogenous mixture of vEVs.

**Fig 2:**
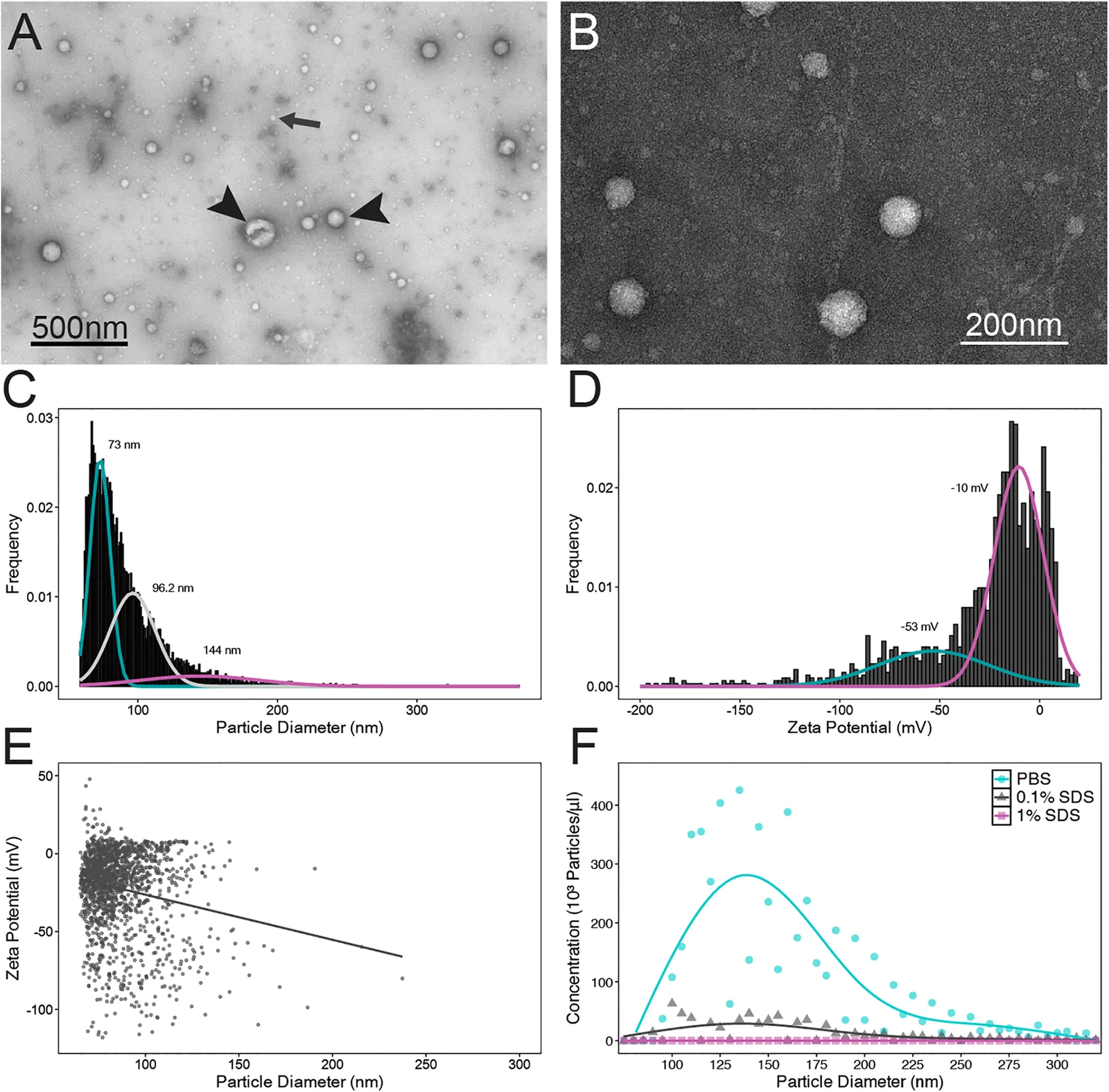
Characterization of vesicles in whole venom. **(A)** Scanning electron micrograph of whole venom. Arrow indicates a non-vesicular extracellular particle; arrowheads indicate differently sized extracellular vesicles. Scale bar is 500 nm. **(B)** Transmission electron micrograph of whole venom. Scale bar is 200 nm. **(C)** Histogram of vesicle diameter measured by TRPS. Mixture model distributions are overlaid, with each distribution mean indicated. **(D)** Histogram of vesicle zeta potential measured by TRPS. Mixture model distributions are overlaid, with each distribution mean indicated. **(E)** Scatter plot of vesicle diameter vs zeta potential with calculated linear regression line. **(F)** Scatter plot of vesicle diameter vs concentration following each of the indicated detergent treatments, with calculated general additive model regression curves.

To characterize *G. hookeri* vEVs, we used tunable resistive pulse sensing (TRPS) to first measure vesicle size and concentration. The distribution of measured diameters (Fig 2C) further supports our conclusion that *G. hookeri* vEVs display size polydispersity. Mixture modeling on this putatively multimodal size distribution predicted three distinct vEV subpopulations, and allowed us to estimate the mean (µ), standard deviation (σ), and weight (or percentage of observations; λ) for each. The most abundant subpopulation (λ = 46.1% of measured vesicles) had a mean diameter (expressed as µ ± σ) of 72.9 ± 7.2 nm, a second subpopulation (λ = 41.9% of measured vesicles) with a mean diameter of 96.2 ± 16.0 nm, and a third subpopulation (λ = 12.1% of measured vesicles) with a mean diameter of 144.5 ± 41.9 nm.

TRPS was next used to measure the zeta potential (or surface charge) of *G. hookeri* vEVs, and we observed a wide range of measured zeta potentials within the population (Fig 2D). Mixture modeling supports the existence of two subpopulations, the first of which has a mean zeta potential of -10.4 ± 12.3 mV (λ = 68.2% of measured vesicles), and a second subpopulation with a mean zeta potential of -53.2 ± 27.5 mV (λ = 24.6% of measured vesicles), with 7.2% of vesicles unaccounted for by these modeled distributions. To explore the relationship between size and surface charge, we plotted vesicle diameter vs zeta potential of vEVs and calculated the linear regression line (Fig 2E). Correlation analysis suggests a weak, yet significant, negative correlation between size and zeta potential (Pearson’s r = -0.19, p = 6.4x10^-15^), suggesting that larger vesicles may have increasingly negative surface charge. However, the scatter plot (Fig 2E) and small Pearson’s r suggest that the relationship between vesicle size and zeta potential is likely more complex.

To characterize the composition of the vEVs, we tested their sensitivity to detergent [32]. We incubated whole venom with increasing concentrations of sodium dodecyl sulfate (SDS), a strong ionic detergent that would be predicted to disrupt vesicle lipid organization, and analyzed vesicle diameter and concentration by TRPS. PBS control treated venom shows the expected size distribution (Fig 2F, cyan). Incubation with 0.1% SDS led to a noticeable decrease in the abundance of vEVs of all sizes (Fig 2F, charcoal). Finally, incubation with 1% SDS abolished nearly all vesicles found in *G. hookeri* venom (Fig 2F, magenta). These findings are consistent with our hypothesis that *G. hookeri* venom contains lipid-enclosed vEVs.

### Density gradient ultracentrifugation resolves *G. hookeri* venom into distinct fractions

To resolve the complex composition of *G. hookeri* venom and the vEV population, we used discontinuous 10–50% sucrose density gradient fractionation to separate whole venom. To test the effectiveness of gradient fractionation for producing unique subpopulations of venom proteins, we performed sodium dodecyl sulfate-polyacrylamide gel electrophoresis (SDS-PAGE) and mass spectrometry proteomic analysis on proteins purified from each fraction to determine the protein composition. By SDS-PAGE, whole venom (Fig 3A, WV) shows a complex banding pattern, which is simplified within each fraction. The venom fractions also display discrete banding patterns, suggesting distinct protein contents (Fig 3A, F5-F1). We mapped mass spectrometry peptides onto the *G. hookeri* transcriptome, and identified 98 proteins in F1, 128 proteins in F2, 118 proteins in F3, 114 proteins in F4, and 94 proteins in F5 (S1 Table). While we observe considerable overlap between fractions, each fraction does have unique members (Fig 3B), in agreement with the SDS-PAGE data (Fig 3A) and suggesting that there is a potential basis for vesicle or fraction specific functions.

**Fig 3:**
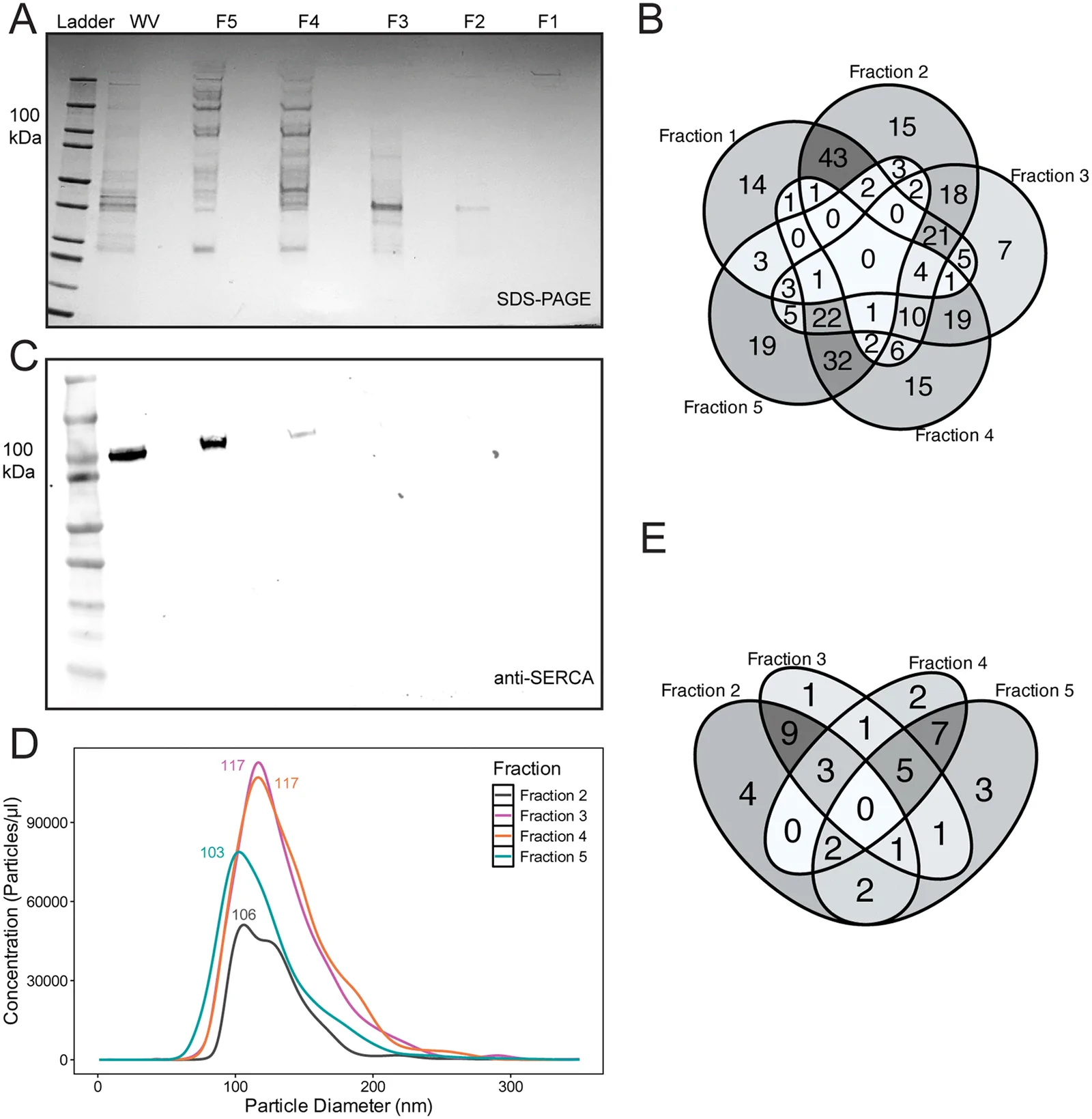
Sucrose gradient fractionation of *G. hookeri* venom. **(A)** SDS-PAGE analysis of whole venom (WV) and each indicated fraction. **(B)** Venn diagram of fraction membership of all proteins identified by mass spectrometry. **(C)** Anti-SERCA Western blot analysis of whole venom following SDS-PAGE as shown in **(A)**. **(D)** Distribution of vesicle diameter by concentration within each indicated fraction as measured by nanoparticle tracking analysis. **(E)** Venn diagram of fraction membership of small EV biomarker proteins identified by mass spectrometry.

Because we hypothesize that venom SERCA is likely found in vEVs, we performed Western blot analysis to determine which venom fraction contains SERCA. The Western blot shows SERCA in whole venom as expected (Fig 3C, WV). We also observe that SERCA is predominantly found in the most dense fraction (F5), with a small amount of protein also visible in F4, in agreement with our mass spectrometry data, in which SERCA (Transcript Acc# GAIW01011364.1) was found in F5 (Fig 3C; S1 Table). This suggests that at least some vEVs are found in F5, which would be unexpected given that F5 represents the 50% sucrose fraction with a density of 1.23 g/mL [33]. Typical extracellular vesicles, including exosomes and microvesicles, have densities in the range of 1.08-1.19 g/mL [34], which would better correspond to fractions F2-F4 (1.08-1.18 g/mL) in our gradient [33]. To test whether vEVs were also found at these densities, we used nanoparticle tracking analysis (NTA) to analyze vEVs across all fractions (Fig 3D). We could not detect vesicles in F1, but vEVs were observed in all other fractions. Interestingly, F2 and F5 had a similar predominant size peak although the overall curves were differently shaped suggesting a different underlying distribution of sizes. F2 and F5 were separable from F3 and F4, which showed identical peak sizes and remarkably similarly shaped curves. Based on these observations, we hypothesize that vEVs are found at distinct densities, and that these between-fraction differences are not due solely to size, given the similarities between vEVs found in F2 and F5, which represent dissimilar densities (1.08 g/mL and 1.23 g/mL; Fig 3D).

Our mass spectrometry data suggest that vEV biogenesis may be conserved with that observed for small EVs in other species. We found a total of 40 proteins in fractions 2–5 that have homology to core small EV proteins identified from human samples (S2 Table) [32,35]. These proteins are considered biomarkers of small EV identity, and include cytoskeletal proteins, enzymes, and protein chaperones, among others (S2 Table) [35]. We found that these small EV biomarker proteins are distributed across the vEV containing fractions, with considerable overlap between fractions (Fig 3E).

We additionally found that the molecular weight and isoelectric point of venom proteins increase with increasing density, although a wide range of values for each of these protein characteristics are found in each fraction (S3A-B Fig). The molecular weight data determined from our mass spectrometry results (S3A Fig) is generally in agreement with our fraction specific SDS-PAGE protein banding patterns (Fig 3A), which also illustrate a trend toward larger proteins in F4 and F5.

### Biophysical characterization of vEVs suggests discrete vesicle populations

To better understand the relationship between fractions, we performed in-depth imaging and biophysical characterization. Based on our NTA data (Fig 3D), we first focused on the relationship between F2 and F5. Negative stain TEM revealed that the vEV contents of F2 are largely monodisperse in size and display a consistent round morphology (Fig 4A). Morphologically similar vEVs are also observed in F5 (Fig 4B), however, F5 also contains a class of larger vEVs with a distinct “kidney bean” shaped morphology (Fig 4C). Following observation of this atypical morphology, we repeated our negative stain TEM characterization using unfixed samples to avoid possible morphology artefacts associated with fixation (Kellenberger et al., 1992), and found that the “kidney bean” morphology is prevalent regardless of fixation (S4 Fig).

**Fig 4:**
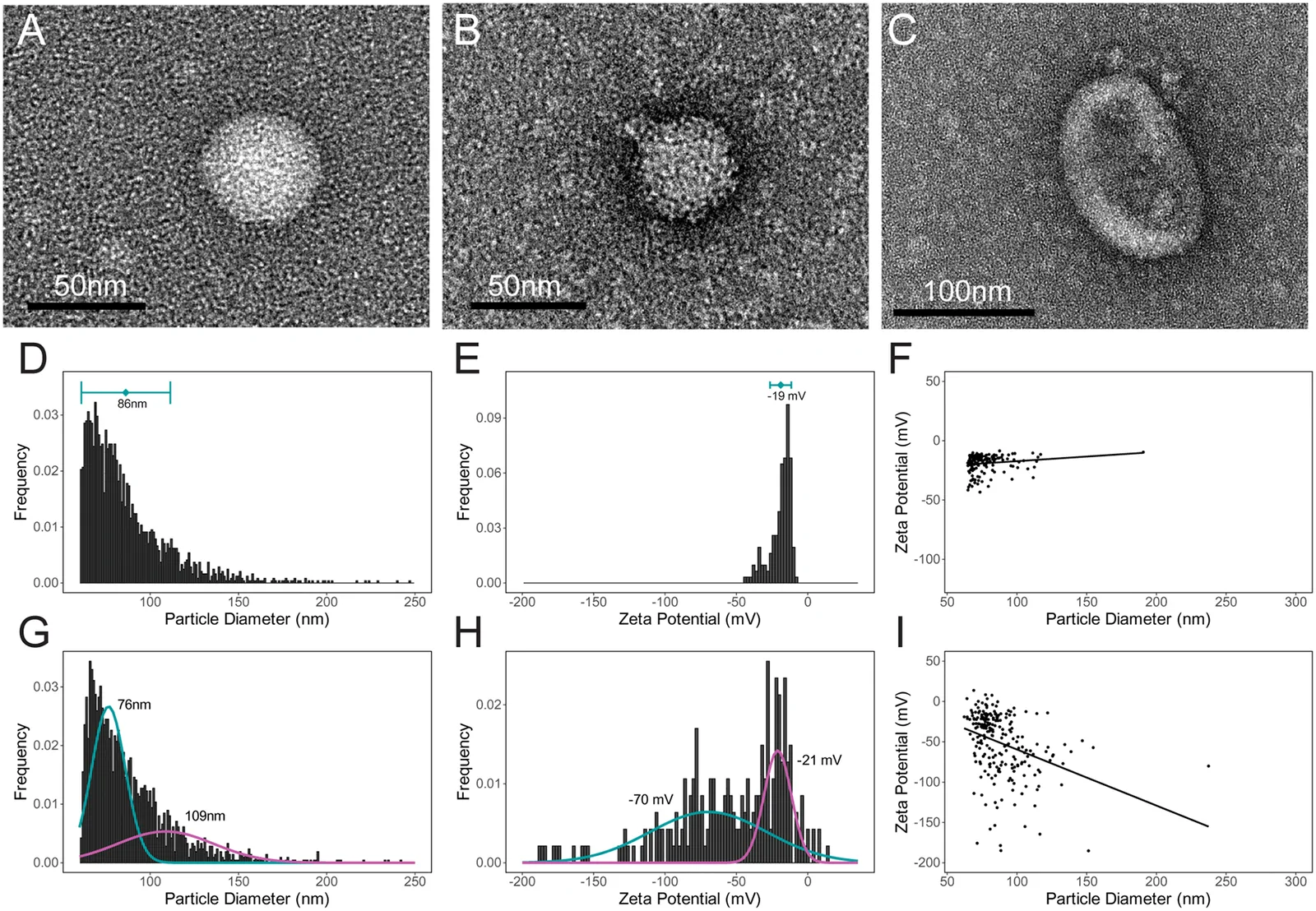
Biophysical characterization of fraction F2 and F5 vEVs. **(A-C)** Transmission electron micrographs of vesicles from F2 (A) and F5 **(B-C)**. Scale bars are 50 nm (A-B) and 100 nm **(C)**. **(D)** Histogram of F2 vesicle diameter measured by TRPS with the mean ± standard deviation of the vesicle population indicated. **(E)** Histogram of F2 vesicle zeta potential measured by TRPS with the mean ± standard deviation of the vesicle population indicated. **(F)** Scatter plot of F2 vesicle diameter vs zeta potential with calculated linear regression line. **(G)** Histogram of F5 vesicle diameter measured by TRPS. Mixture model distributions are overlaid, with each distribution mean indicated. **(H)** Histogram of F5 vesicle zeta potential measured by TRPS. Mixture model distributions are overlaid, with each distribution mean indicated. **(I)** Scatter plot of F5 vesicle diameter vs zeta potential with calculated linear regression line.

We performed TRPS in order to more thoroughly characterize the composition of each fraction. In agreement with the TEM imaging results, F2 has a relatively monodisperse population of vEVs, and mixture modeling of F2 data reveals a single vesicle population (Fig 4D-F). Vesicles in F2 have a mean diameter of 86.2 ± 25.2 nm (Fig 4D), and a mean zeta potential of -19.0 ± 7.4 mV (Fig 4E). The linear regression line of the size vs zeta potential plot (Fig 4F) shows a weak positive correlation between these properties (r = 0.16, p = 9.3x10^−5^).

Conversely, TPRS analysis of the vEVs in F5 presents evidence for polydispersity, in agreement with the TEM imaging results. We performed mixture modeling of vEV characterization data to resolve this mixed population. Our modeling suggests that F5 contains two populations of vEVs based on diameter (Fig 4G); the first subpopulation (λ = 63.6% of the vesicles measured) has a mean diameter of 76.4 ± 9.5 nm, and the second subpopulation (λ = 36.3% of the vesicles measured) has a mean diameter of 108.7 ± 27.2 nm. This bimodal distribution is also reflected in our measurements of zeta potential, with the mixture model again suggesting two populations (Fig 4H). The first subpopulation (λ = 66.0% of the vesicles measured) has a mean zeta potential of -69.7 ± 40.9 mV, and the second subpopulation (λ = 34.0% of the vesicles measured) has a mean zeta potential of -21.1 ± 9.5 mV. Linear regression of the diameter vs zeta potential plot (Fig 4I) suggests that there is a moderate negative correlation (r = -0.34, p = 2.2x10^−16^) with the larger population of vEVs having a more negative zeta potential.

Our NTA data (Fig 3D) further suggest that F3 and F4 likely contain highly similar vEV populations. Negative stain TEM imaging supports this hypothesis with both fractions showing largely homogenous populations of similarly sized, round vesicles (Fig 5A-B). TRPS analysis bears out the size similarity, with F3 having a mean diameter of 96.5 ± 32.9 nm and F4 having a mean diameter of 93.4 ± 31.6 nm (Fig 5C-D). However, characterization of zeta potential suggests that F3 and F4 are distinct despite this size similarity (Fig 5E-F). F3 has a mean zeta potential of -12.5 ± 26.9 mV. Despite the homogeneity of size and morphology, F4 appears to have a bimodal distribution of zeta potentials; by mixture modeling, the first subpopulation (λ = 51.1% of the vesicles measured) has a mean zeta potential of -65.1 ± 47.3 mV, and the second subpopulation (λ = 48.9% of the vesicles measured) has a mean zeta potential of -22.4 ± 10.6 mV (Fig 5F). Linear regression of the diameter vs zeta potential plots suggests that there is no correlation (r = -0.031, p = 0.062) between size and charge in F3 (Fig 5G), and a weak negative correlation (r = -0.3, p = 2.2x10^−16^) in F4 (Fig 5H).

**Fig 5:**
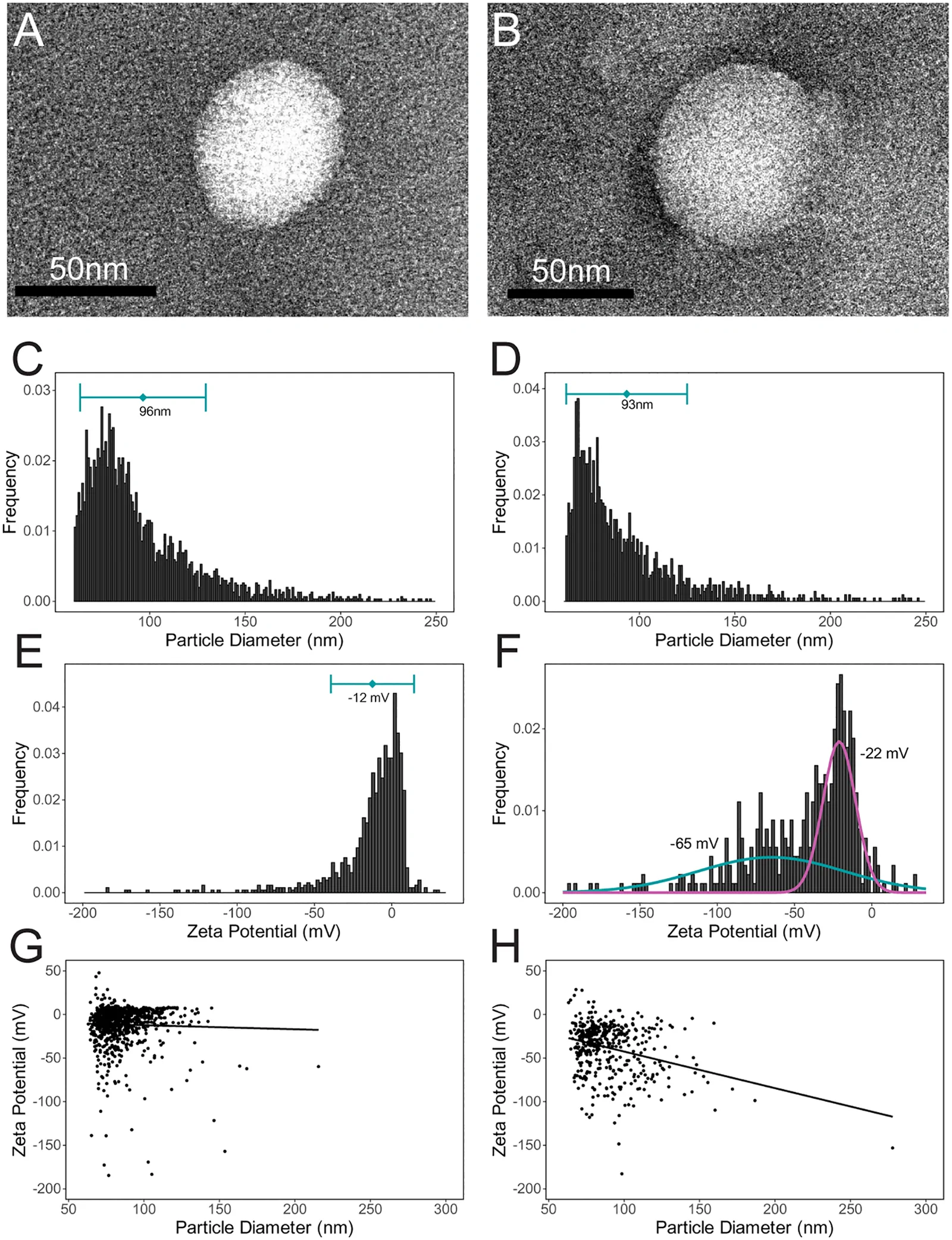
Biophysical characterization of fraction F3 and F4 vEVs. **(A-B)** Transmission electron micrographs of vesicles from F3 (A) and F4 **(B)**. Scale bars are 50 nm. **(C)** Histogram of F3 vesicle diameter measured by TRPS with the mean ± standard deviation of the vesicle population indicated. **(D)** Histogram of F4 vesicle diameter measured by TRPS with the mean ± standard deviation of the vesicle population indicated. **(E)** Histogram of F3 vesicle zeta potential measured by TRPS with the mean ± standard deviation of the vesicle population indicated. **(F)** Histogram of F4 vesicle zeta potential measured by TRPS. Mixture model distributions are overlaid, with each distribution mean indicated. **(G)** Scatter plot of F3 vesicle diameter vs zeta potential with calculated linear regression line. **(H)** Scatter plot of F4 vesicle diameter vs zeta potential with calculated linear regression line.

### Mass spectrometry identifies the protein composition of *G. hookeri* venom fractions

Our observation of *G. hookeri* vEVs suggests that they serve to transport venom proteins into host cells following infection as has been observed in other parasitoid species. However, in these other species, venom proteins are found within a single vesicle type [23,27], rather than the heterogeneous population of vEVs we have characterized in *G. hookeri*. We hypothesize that the distinct vesicle populations we observe across venom fractions may contain distinct protein cargos. To test this, we used the KofamKOALA algorithm to map our mass spectrometry identified venom proteins onto known proteins for functional prediction [36]. Our protein identification results were then compared to the Kyoto Encyclopedia of Genes and Genomes (KEGG) database by the KEGG Mapper tool [37]. This analysis revealed potential functions for each of the venom fractions as represented by membership in KEGG pathway modules (Table 1) [38]. Most of these putative functions are related to metabolism, including the initial and elongation steps of fatty acid biosynthesis (found in distinct fractions), and glycogen degradation and beta-oxidation, both of which are catabolic processes linked to energy production [39].

**Table 1:** KEGG Modules identified among *G. hookeri* venom proteins.

Function	Proteins	Fraction(s)
Actin binding proteins	Profilin [K05759]; Cofilin [K05765]; Tropomyosin I [K10373]	1, 2
Fatty acid biosynthesis, initiation [M00082]	Fatty acid synthase [EC:2.3.1.85], acetyl-CoA carboxylase [EC 6.4.1.2]	5
Fatty acid biosynthesis elongation [M00083]	Fatty acid synthase [EC:2.3.1.85]	3, 4
Glycogen degradation [M00855]	Glycogen phosphorylase [EC:2.4.1.1]; Glycogen debranching enzyme [EC:2.4.1.25;3.2.1.33]; Phosphoglucomutase [EC:5.4.2.2]	4
Beta-oxidation [M00087]	Acyl-CoA dehydrogenase [EC:1.3.8.7]; Acetyl-CoA acyltransferase [EC:2.3.1.16]; Enoyl-CoA hydratase [EC:4.2.1.17]	4, 5

The observed overlap in protein content between fractions (Fig 3B) raises the possibility that vEVs with similar physical properties may also have similar cargo. To test this idea, we analyzed our mass spectrometry data to identify proteins that are specific to F2 and F5 or F3 and F4. F2 and F5 have minimal overlap of protein content, with only 10 proteins in common and 3 proteins that are specific to F2 and F5 and excluded from the other fractions. These differences in protein content potentially underlie the observed density difference. On the other hand, F3 and F4 show a great deal of similarity of protein content. In total, 57 proteins are shared between these fractions, and among these we identified 19 proteins that are specifically shared between F3 and F4 and excluded from the other fractions (Table 2). These F3/F4 specific proteins include both predicted metabolic enzymes and proteins predicted to be involved in cell signaling, suggesting that vEV cargos may play multiple functions in host cells.

**Table 2:** Proteins specific to fractions F3/F4 predicted by fraction-specific mass spectrometry.

Protein	Transcript Accession
Leucine rich repeat protein [COG4886]	GAIW01007069.1
Pyruvate dehydrogenase E1 component subunit alpha [EC:1.2.4.1]	GAIW01010504.1
Reticulon-1 [K20721]	GAIW01010536.1
Alpha-glucosidase [EC:3.2.1.20]	GAIW01010903.1
NADP + -dependent farnesol dehydrogenase [EC:1.1.1.216]	GAIW01011046.1
Trehalose 6-phosphate phosphatase [EC:3.1.3.12]	GAIW01011239.1
Presequence protease [EC:3.4.24.-]	GAIW01011585.1
Troponin I [P36188.3]	GAIW01012017.1
Clavesin [K28049]	GAIW01012229.1
Juvenile hormone binding protein [pfam06585]	GAIW01012823.1
Ubiquitin-activating enzyme E1 [EC:6.2.1.45]	GAIW01012873.1
Ezrin-moesin-radixin 1 [K26219]	GAIW01013187.1
Nidogen [K06826]	GAIW01019606.1
Glucose-6-phosphate isomerase [EC:5.3.1.9]	GAIW01021188.1
Aconitate hydratase [EC:4.2.1.3]	GAIW01021311.1
Transcriptional activator protein Pur-alpha [K21772]	GAIW01021479.1
Dipeptidyl-peptidase III [EC:3.4.14.4]	GAIW01021615.1
Unannotated	GAIW01022664.1
Unannotated	GAIW01023502.1

### vEVs gain entry into host plasmatocytes

We hypothesize that vEVs may serve as venom protein transport mechanisms. If this is the case, then we would predict that vEVs would interact with, and potentially enter into, host cells. To assess this, we stained whole venom with ExoSparkler (Exo-Venom), a lipophilic dye that enables tracking of vesicle internalization by fluorescence microscopy [40]. PBS alone was incubated with ExoSparkler (Exo-PBS) to control for any background staining or dye carryover following dye removal. Exo-Venom and Exo-PBS were then incubated with primary hemocyte cultures established from *hop[Tum]* third instar larvae. *hop[Tum]* is a gain of function mutation in the JAK-STAT pathway which leads to the ectopic production of lamellocytes [41], allowing us to assess vEV interactions with both plasmatocytes and lamellocytes. After 40 minutes of incubation with Exo-Venom or Exo-PBS, cells were fixed and imaged to assess ExoSparkler localization (Fig 6A-B; S5 Fig).

**Fig 6:**
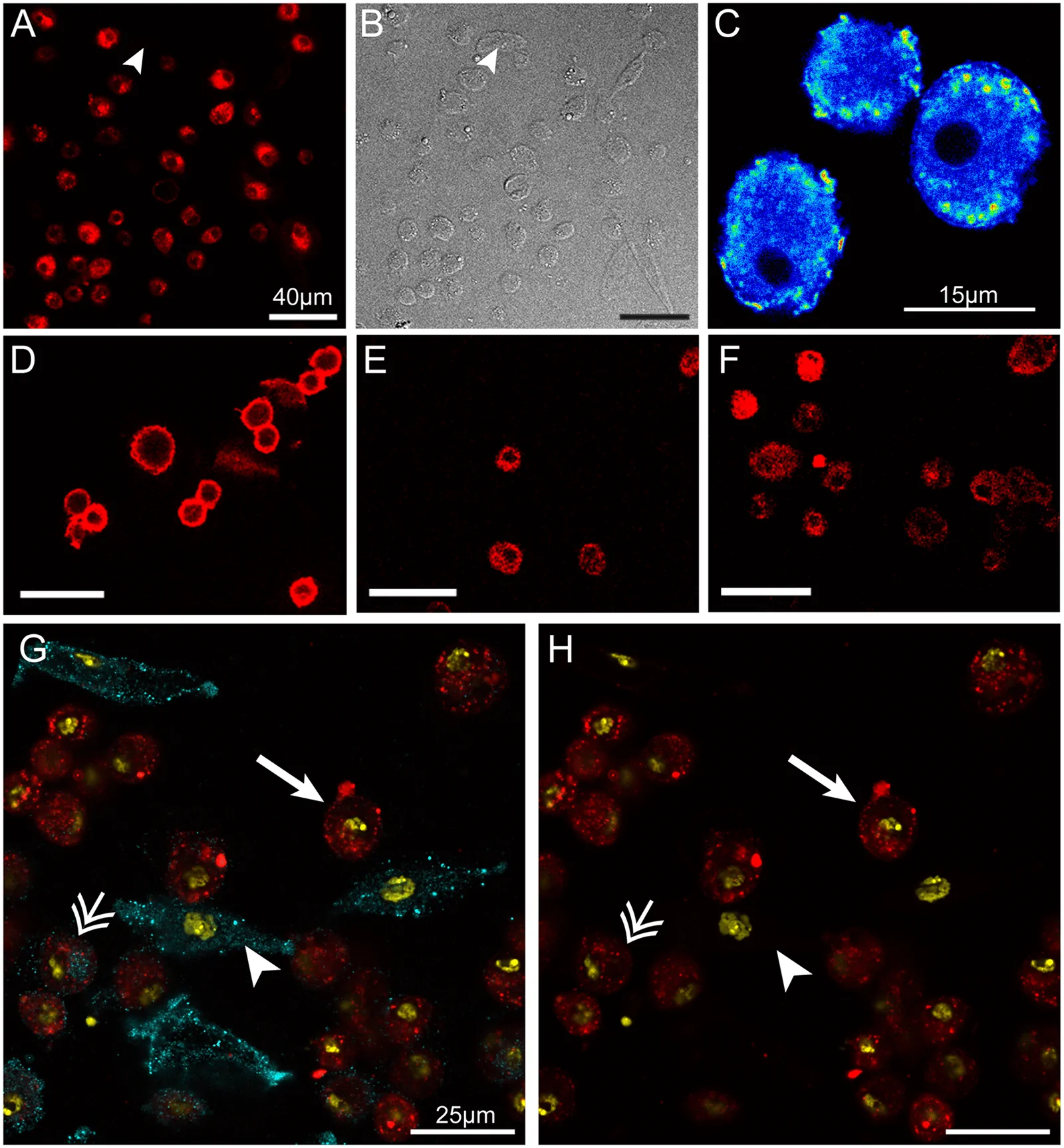
Analysis of ExoSparkler labeled vEV (Exo-Venom) entry into host cells. Scale bars are 40 µm in A-B,D-F, 15 µm in C, and 25 µm in G-H. (A-B) *hop[Tum]* hemocyte primary cultures incubated with Exo-Venom, confocal micrograph taken at 40X magnification (A) shown with paired brightfield image **(B)**. Arrowheads indicate unstained lamellocytes. See S5 Fig for additional representative fields and Exo-PBS control. **(C)** Confocal micrograph of Exo-Venom treated *hop[Tum]* hemocyte primary cultures taken at 63X magnification with intensity values quantized using a 16-color LUT. Warmer colors indicate higher intensity values. S1 Movie shows successive Z-series images. **(D-F)** Time course of *w[1118]* hemocyte primary cultures. For time course analysis, cells were fixed following 5 minutes **(D)**, 15 minutes **(E)**, or 40 minutes (F) of incubation with Exo-Venom and imaged by confocal microscopy at 40X magnification. **(G-H)** Confocal micrograph of *hop[Tum]* hemocyte primary cultures treated with Exo-Venom and stained with anti-Atilla antibody to mark lamellocytes, taken at 63X magnification. Exo-Venom signal is shown in red **(G-H)**, anti-Atilla stain in cyan **(G)**, and DAPI in yellow **(G-H)**. The arrow indicates an Atilla negative plasmatocyte showing Exo-venom uptake. The arrowhead indicates an Atilla positive mature lamellocyte showing an absence of Exo-venom signal. The double arrowhead indicates an intermediate lamellocyte, with minimal Atilla staining and Exo-venom signal.

We observed that Exo-Venom incubated plasmatocytes had high levels of fluorescence, both at the membrane and within the cytoplasm (Fig 6A-C; S5A-B Fig), although the signal appears to be excluded from the nucleus (Fig 6A,C; S5A Fig). This suggests that labeled vEVs are able to gain entry into plasmatocytes, and quantized fluorescence intensity data reveal that labeled particles are found in discrete puncta throughout the cytoplasm, with a greater intensity near the cell cortex (Fig 6C; S1 Movie). Fluorescence was largely undetectable in Exo-PBS treated hemocytes (S5C-D Fig), suggesting that our dye cleanup was successful and that the fluorescence entry into host cells is specific to ExoSparkler labeled vEVs.

The apparent localization of ExoSparkler within the cytoplasm of plasmatocytes is consistent with the hypothesis that vEVs may be gaining entry into host cells. To better understand this process, we performed a time course experiment to track the interaction. We incubated primary plasmatocyte cultures from *w[1118]* larvae with Exo-Venom for 5, 15, and 40 minutes before fixation and imaging. We observed that at the earliest time point, fluorescence signal was localized nearly exclusively to the plasma membrane (Fig 6D). After 15 minutes of incubation, cytoplasmic fluorescence was observed (Fig 6E), and by 40 minutes nearly all of the signal was internalized into the cytoplasm (Fig 6F). These findings suggest that vEVs gain entry into host plasmatocytes, and that this process is mediated by a stepwise mechanism.

We did not observe detectable levels of fluorescence in lamellocytes (Fig 6A-B, indicated by arrowheads), suggesting that *G. hookeri* vEVs may specifically target plasmatocytes in agreement with previous data on *G. hookeri* venom SERCA activity [14]. To test the specificity of vEV entry into different hemocyte subtypes, we incubated labeled vEVs with primary hemocytes established from *hop[Tum]* third instar larvae and stained with the lamellocyte-specific marker Atilla/L1 [42]. We found that vEVs readily enter into host plasmatocytes (arrow in Fig 6G-H), but are excluded from mature lamellocytes as defined by elevated Atilla expression (arrowhead in Fig 6G-H). Intermediate lamellocytes, as defined by plasmatocyte-like morphology and weak Atilla expression [43], show minimal vEV uptake (double arrowhead in Fig 6G-H).

Finally, to test if vEVs are required for venom SERCA activity, we isolated F5 from venom gradients. The isolated F5 venom was divided into three groups: (1) untreated F5 venom served as a positive control for venom activity, (2) heat treated F5 venom that was exposed to 90°C for 5 minutes to heat denature any proteins in the sample served as a control for suppressed venom activity, and (3) sonicated F5 venom that was treated with water bath sonication at full power for 15 minutes to disrupt vesicle integrity served as a test of the requirement for intact vEVs for venom SERCA activity. We then incubated primary plasmatocyte cultures expressing the genetically encoded *GCaMP3* calcium sensor under control of the *Cg-GAL4* driver [14] with untreated F5 samples, heat denatured F5 samples, and sonicated F5 samples and measured *GCaMP3* fluorescence intensity in comparison with control PBS (no venom) treated cells. We found that incubation with untreated F5 led to a significant decrease in intracellular calcium levels compared to PBS control, as measured by *GCaMP3* fluorescence intensity (Fig 7), in agreement with our discovery of SERCA as an F5 component (Fig 3C; S1 Table). As expected, prior heat denaturation of F5 venom significantly inhibited its ability to alter *GCaMP3* fluorescence intensity (Fig 7). We further found that the activity of sonicated F5 was indistinguishable from heat denatured F5 or PBS control (Fig 7), suggesting that vesicle integrity is required for the venom effect on *GCaMP3* fluorescence intensity.

**Fig 7:**
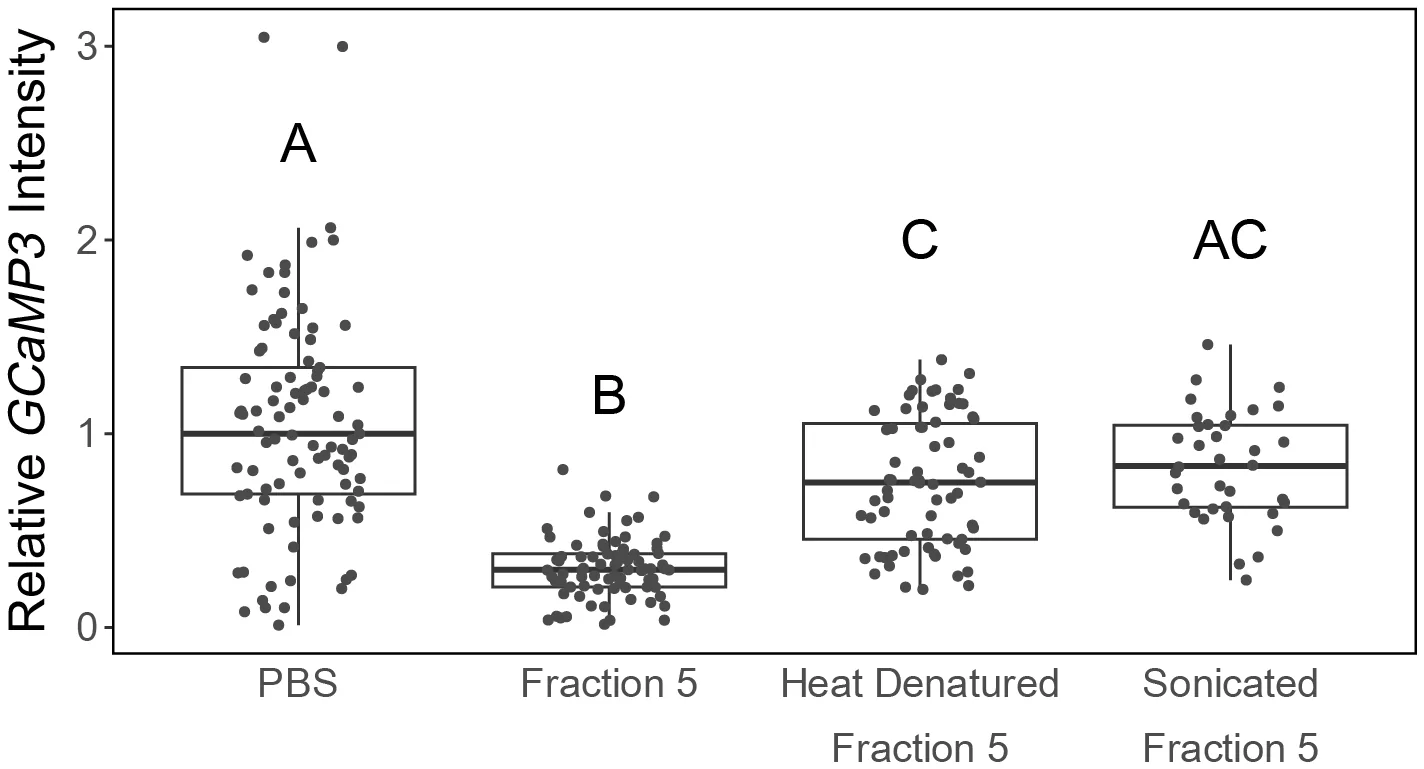
Analysis of vEV function in host cells. Box plot of relative *GCaMP3* fluorescence intensity, normalized to PBS control, in primary plasmatocyte cultures isolated from *Cg:GCaMP3* larvae. Cells were treated with PBS, purified untreated F5, heat denatured F5, or sonicated F5, as indicated. Letters indicate confidence groups assigned by Dunn’s *post hoc* test.

## Discussion

To test our hypothesis that *G. hookeri* venom proteins, such as the large hydrophobic venom SERCA, are transported into host cells via extracellular vesicles, we characterized the composition of *G. hookeri* venom through biophysical, cell biological, and biochemical approaches. Our findings reveal that *G. hookeri* venom contains a heterogenous population of small vEVs, which gain entry into *D. melanogaster* immune cells. It seems likely that at least one of these vesicle types serves to transport venom SERCA into host cells given that disruption of vesicle integrity blocks the anti-calcium effect of *G. hookeri* venom equivalently to venom heat denaturation (Fig 7). Our finding that vesicle entry targets host plasmatocytes and largely excludes lamellocytes further aligns with our previous findings that *G. hookeri* venom SERCA activity is specific to plasmatocytes during infection [14].

To further characterize vEV cargo, we performed fraction-specific mass spectrometry, and identified unique proteins in each fraction, along with proteins found across multiple fractions. Similar to previous reports [23,44], we identified additional venom proteins after optimizing our protein purification approach to recover vesicle proteins in comparison with our previous *G. hookeri* venom mass spectrometry approach that assumed venom was composed of soluble proteins and complexes [14]. Our mass spectrometry results suggest that vEV cargos likely contain non-redundant subsets of venom proteins with a wide range of possible functions. This leads to us hypothesize that each vEV type may play a distinct functional role in infection. For instance, SERCA containing vEVs in F5 likely enhance *G. hookeri* virulence via suppression of host immune signaling, whereas F4 vEVs contain proteins homologous to enzymes involved in glycogen degradation and beta-oxidation (Table 2), suggesting that they may play a role in manipulating host energy stores.

The release of small EVs is a characteristic of infection across a wide range of parasite and parasitoid species [23,25,27]. Previous research has revealed that parasite-derived small EVs enable the transfer of virulence factors into host cells and contribute to parasite infection success. The use of small EVs is broadly conserved, and EV release has been observed from diverse phyla including gram-negative and gram-positive bacteria [45], protozoan parasites such as *Leishmania*, *Plasmodium falciparum*, *Trichomonas vaginalis*, *Giardia intestinalis* [24,46,47], the parasitic euglenoid *Trypanosoma cruzi* [24], the parasitic alveolate *Toxoplasma gondii* [25], and macroparasites including parasitic helminths [48,49], and parasitoid wasps such as *L. boulardi* and *L. heterotoma* [23,27].

The protein cargos isolated from *G. hookeri* vEVs show a surprising conservation with 40 previously identified protein biomarkers of small EV identity from human samples found in vEV containing venom fractions. The EVs from other parasite species also show a partial conservation of biomarker proteins with these canonical small EVs [24,25], suggesting that despite putative differences in EV biogenesis [45], parasite-derived EVs may represent an evolutionarily related subset of the eukaryotic EV repertoire.

Our biophysical characterization of venom vesicles highlights potential differences in vesicle usage between *G. hookeri* and other Figitidae with described vEVs (Table 3). *L. heterotoma* vEVs, formerly known as virus-like particles, compose a generally homogenous population of large (300 nm) vesicles with a distinct knob-like morphology [28,29]. The density of these extracellular vesicles has not been determined; however they are purified as a single fraction from a Nycodenz gradient, suggesting that the density is likewise uniform [23]. The vEVs from *L. boulardi*, commonly known as venosomes, are 100–200 nm in diameter, and are also found in an aggregated state with aggregates having a total size of 400 nm - 1 µm [27]. There is disagreement over the morphology of these venosomes, with reports differing between a stellate or round morphology [26,27]. It is unclear from previous reports whether these morphological differences represent unique vesicle subpopulations within *L. boulardi* venom or if they may be artefacts of sample preparation or imaging. Again, although the density has not been determined, these venosomes are purified as a single fraction at 25–30% Nycodenz [27]. In contrast, *G. hookeri* have as many as 6 separable populations of venom vesicles when diameter, morphology, zeta potential, and density are taken into account (Table 3). These populations include low-density small vEVs (F2 vesicles), and two populations of medium-density small vEVs, that are separable by zeta potential (F3 and F4b vesicles with mean zeta potentials between -12 mV and -21 mV, and F4a vesicles with a mean zeta potential of -65 mV). Finally, we have identified two populations of high-density vEVs that otherwise differ by size, morphology, and zeta potential (F5a and F5b vesicles, Table 3).

**Table 3:** Comparison of venom vesicle morphology and biophysical characterization of putative vEV classes within *G. hookeri* venom and across parasitoid species.

Species	Vesicle Size Range	Vesicle Zeta Potential	Vesicle Density	Vesicle Morphology
*G. hookeri* F2	86.2 ± 25 nm	-19.0 ± 7 mV	1.08 g/mL	Small, round
*G. hookeri* F3	96.5 ± 33 nm	-12.5 ± 27 mV	1.13 g/mL	Small, round
*G. hookeri* F4a	93.4 ± 32 nm	-65.1 ± 47 mV	1.18 g/mL	Small, round
*G. hookeri* F4b	93.4 ± 32 nm	-22.4 ± 11 mV	1.18 g/mL	Small, round
*G. hookeri* F5a	76.4 ± 9 nm	-21.1 ± 10 mV	1.23 g/mL	Small, round
*G. hookeri* F5b	108.7 ± 27 nm	-69.7 ± 41 mV	1.23 g/mL	Large, kidney bean shaped
*L. heterotoma* Extracellular Vesicles	300 nm	N.D.	N.D. (single fraction in Nycodenz gradient)	Large, knob-like
*L. boulardi* Venosomes	100 - 200 nm (Aggregates: 400 nm – 1µm)	N.D.	N.D. (single fraction at 25–30% Nycodenz)	Large, stellate or round

Density values for sucrose determined from [33]; data on *L. heterotoma* extracellular vesicles from [23,28,29]; data on *L. boulardi* venosomes from [26,27]; N.D. not determined.

It is important to note that methodology differences between TRPS and NTA can account for the slight differences in vesicle diameter that we measured between experiments [50–52]. TRPS uses an electrical approach in which sensing a particle causes a resistive pulse. The resistive pulse height is proportional to particle volume, and diameter is calculated from this measurement [53]. In contrast, NTA uses optical measurement of the hydrodynamic diameter, which is influenced by particle hydration and diffusion behavior, resulting in slightly larger diameter measurements [51]. However, the relative differences between fractions are very consistent between these disparate methods, and our TRPS data match well with sizes estimated from TEM imaging, suggesting that our presented vesicle diameter measurements likely provide an accurate reflection of the *G. hookeri* venom composition.

Our combined results from mass spectrometry and SDS-PAGE suggest that the more dense fractions contain larger proteins, although in both cases, we discovered a large range of protein sizes in each fraction. Because density is not merely a product of size, this suggests that larger proteins may be more likely to be found associated with higher density particles, such as vesicles. Similarly, while there are differences in the mean protein isoelectric point across fractions, these differences are not predictive of the vesicle zeta potentials measured. Because zeta potential reflects the surface potential of the vesicle, it is likely driven primarily by lipid composition with contributions from the proteins found on the surface of the vesicle membrane [54,55]. Additionally, our data show a small but significant negative correlation between vesicle size and zeta potential, potentially revealing a relationship between these properties that is also strongly influenced by the other factors discussed.

More generally, our findings suggest that the characterization of newly discovered vesicle populations should include more than EM analysis or size determination in isolation (Table 3) [32]. We uncovered morphologically similar vesicles that are distinct when compared by other parameters. For instance, F2 vEVs and F5a vEVs have similar sizes, morphology, and zeta potential, but display dramatically different densities and have minimal overlap in protein content. Likewise, although F4 vEVs appear homogeneous in terms of morphology, size, and density, F4 contains two subpopulations of vEVs with drastically different zeta potentials, suggesting the potential presence of different functional populations of vesicles within this fraction.

Our characterization of *G. hookeri* venom vesicles provides insight into the intricate virulence strategies used by parasitoid wasps to overcome host defenses, and may lead to further discoveries in the principles underlying protein packaging and transport in membrane enclosed vesicles.

## Materials and methods

### Insects

Flies were maintained on standard Drosophila medium of cornmeal, yeast, and molasses. The wild type controls *w[1118]* (RRID: BDSC_5905) and *Canton-S* (RRID: BDSC_64349), the *Cg-GAL4* driver (RRID: BDSC_605136), the genetically encoded calcium sensor *UAS-GCaMP3* (RRID: BDSC_ 32116), and the *hop[Tum]* (RRID: BDSC_8492) mutant *Drosophila melanogaster* strains were obtained from the Bloomington Drosophila Stock Center (Bloomington, IN) (RRID: SCR_006457). The *Drosophila yakuba* strain Tai18E2 (RRID: NCBITaxon_7245) was obtained from Kyorin-Fly (Tokyo, Japan). *Ganaspis hookeri* (NCBI: txid2777979) wasps were maintained on *D. yakuba* as previously described [14].

### Venom purification

To extract *G. hookeri* venom, the venom apparatus was dissected from adult female wasps into HALT protease inhibitor (Thermo Fisher Scientific) diluted to 1X in phosphate buffered saline (PBS: 137 mM NaCl, 2.7 mM KCl, 10 mM Na_2_HPO_4_, 1.8 mM KH_2_PO_4_, pH 7.4). Dissected tissues were pelleted by centrifugation at 8,000 x g and homogenized (Pestle Homogenizer, Fisher Scientific) in short pulses of 2 seconds under non-lysing conditions. The homogenized sample was incubated on ice for 5 minutes and centrifuged at 1,000 x g for 10 minutes to pellet cells, cellular debris, and any remaining tissues. Following centrifugation, the supernatant was removed from the pellet and stored at 4°C. We observed venom vesicle aggregation at 4°C by electron microscopy (S2A-B Fig) and TRPS (S2C Fig). Electron microscopy reveals that individual particles are predominantly < 200 nm in diameter, so venom samples were filtered using a 0.22 µm PES syringe filter (SFPES004022N, Membrane Solutions) prior to use. TRPS analysis of unfiltered and filtered venom samples shows a minimal change in the vEV population, and SDS-PAGE analysis demonstrates that venom composition is minimally changed by filtration (S2C-D Fig). See [56] for a step-by-step protocol for venom purification.

### Detergent treatment for vesicle disruption

To test whether venom vesicles are sensitive to detergent disruption, purified venom from 30 *G. hookeri* wasps was incubated with 0.1% SDS (w/v), 1% SDS (w/v), or PBS control for one hour before further TRPS analysis was performed as described.

### Fluorescent vesicle labeling

To label *G. hookeri* venom vesicles, 100 µL of purified venom collected from 50 venom apparatus was combined with 2 µL of ExoSparkler Membrane Labeling dye (Dojindo), resuspended at 1X in 10 µL dimethyl sulfoxide (DMSO, Fisher Scientific), and incubated at 37°C for 30 minutes. A control sample of 100 µL PBS was simultaneously incubated with 2 µL of ExoSparkler at 37°C for 30 minutes. Both venom and control samples were then transferred to the provided ExoSparkler collection tubes and centrifuged at 3,000 x g for 5 minutes at room temperature for the removal of unbound dye. Samples were washed twice with PBS and spun dry at 3,000 x g. Labeled venom and control samples were recovered in 50 µL of PBS.

### Confocal microscopy

Confocal microscopy was performed on Leica SP8 and Leica Stellaris 5 confocal microscopes (Leica Microsystems). The SP8 was used with a HC PL APO 40x/1.10 W motCORR CS2 objective and a HC PL APO 63x/1.40 OIL CS2 objective as indicated in the text, and the Stellaris 5 was mounted on a DMi8 inverted microscope using a HC PL APO 40x/1.10 W motCORR CS2 water immersion objective. Both systems used a white light laser set to the appropriate excitation wavelengths in a room temperature of 19°C.

#### Whole mount venom apparatus imaging.

For whole mount imaging, the venom apparatus was dissected from 10 adult female wasps into PBS. Tissues were fixed in 4% paraformaldehyde in PBS (w/v, Fisher Scientific) for 10 minutes, and permeabilized in PBS + 0.1% Triton-X100 (PBSTx) for 10 minutes. Tissues were then blocked in 5% normal goat serum in PBSTx and stained with the α-SERCA 809–27 antibody [57] diluted 1:1,000 in blocking solution, and FITC-conjugated fluorescent secondary antibody diluted 1:500 in blocking solution (Jackson ImmunoResearch). Venom apparatuses were co-stained with 165nM phalloidin-Alexa Fluor 633 (Molecular Probes) in PBS for 10 minutes. Stained tissues were mounted whole in VectaShield (VectorLabs) and imaged on the SP8 confocal as described above. Z-stack images spanning the depth of the tissue were taken at 40x and 63x magnification as indicated in the text. LAS X Navigator was used to define regions of interest and stitch Z-stack images taken in multiple fields of view. Z-stacks were processed in FIJI [58] for maximum intensity 2D projections, and “Brightest Point” 3D projections using default parameters.

#### Fluorescently labeled vesicle imaging.

To assess venom vesicle entry into *D. melanogaster* plasmatocytes, we incubated venom vesicles fluorescently labeled with ExoSparkler (see above) with primary hemocyte cultures and used confocal microscopy to image vesicle entry. Primary hemocyte cultures were derived from second instar *hop[Tum]* larvae: larvae were rinsed in PBS, and bled in 35 µL of PBS on a dissection slide (Tekdon, Inc) in duplicate. Cells were allowed to become adherent for 10 minutes. PBS was removed and 30 µL of ExoSparkler-venom or ExoSparkler-PBS control were added, and cells were incubated for 40 minutes shielded from light. Solutions were removed and cells were fixed with 4% paraformaldehyde in PBS for 15 minutes (Fisher Scientific), washed in PBS and mounted in VectaShield (VectorLabs). Cells were imaged on the SP8 confocal at 40x magnification as described above.

To better highlight localized differences in fluorescence intensity, a 16-colour LUT was applied to ExoSparkler intensity data in FIJI [58]. This process of quantization remaps the intensity data to 16 color scale bins to highlight localized intensity values and enhance visualization [58,59].

#### Vesicle entry time course imaging.

To investigate the process of venom vesicles entry into host plasmatocytes, primary plasmatocyte cultures were derived from second instar *w[1118]* larvae as described above. Cells were incubated with 30 µL ExoSparkler-venom for 5, 15, or 40 minutes, then fixed in 4% paraformaldehyde in PBS for 15 minutes. Cells were mounted in VectaShield (VectorLabs), and imaged on the SP8 confocal at 40x magnification as described above.

#### Hemocyte immunocytochemistry.

To evaluate venom vesicle specificity for plasmatocytes, hemocytes were isolated from five *hop[Tum]* third-instar larvae in PBS on a dissection slide and allowed to adhere for 10 min. Cells were incubated with ExoSparkler-labeled venom (20 µL) for 45 min, followed by two 5-minute PBS washes. Hemocytes were permeabilized with 0.01% Triton X-100 in PBS (PBS-T) for 5 min and blocked in PBS-T containing 5% BSA for 1 hour. Lamellocytes were labeled using an anti-Atilla primary antibody (1:100 in blocking buffer) [42] for 1 hour, washed twice with PBS-T (5 min each), and incubated with Alexa Fluor 488–conjugated goat anti-mouse IgG secondary antibody (1:500; 1 h). Nuclear DNA was stained with DAPI (1 µg/mL, 5 min). Following two 5-minute PBS washes, cells were mounted in VectaShield and imaged on the Stellaris 5 confocal microscope at 40x magnification as described above.

### Sucrose gradient fractionation

To separate *G. hookeri* venom proteins and vesicles by density, purified venom was subjected to sucrose gradient fractionation through a discontinuous gradient ranging from 10 - 50% (w/v) sucrose. A PBS buffered 66% (w/v) sucrose solution was diluted in PBS to final concentrations of 50%, 40%, 30%, 20%, and 10% on ice. The discontinuous gradient was generated by underlaying progressively more dense solutions in thick-wall polycarbonate tubes (11x34 mm, Beckman Coulter). 100 µL of venom or PBS only control were applied to the top of the sucrose gradient and samples were centrifuged in a swinging bucket rotor (TLS-55, Beckman Coulter) at 120,000 x g for 20 hours at 4°C (Beckman Optima TLX) without braking.

### Sodium Dodecyl Sulfate-Polyacrylamide Gel Electrophoresis (SDS-PAGE)

SDS-PAGE was used to analyze venom protein profiles from sucrose gradient fractionation. Purified venom from 60 female wasps was fractionated by sucrose gradient fractionation protocol. Fractions were dialyzed into 100 µL of PBS using 10K MWCO Slide-A-Lyzer (ThermoFisher) dialysis cups with two room temperature buffer exchanges and an overnight dialysis at 4°C. For SDS-PAGE analysis, 20 µL of venom from each fraction was sonicated at full power using a Branson CPX1800H ultrasonic water bath (Branson Ultrasonics) for 15 minutes, and combined with 4 µL of 6X Laemmli SDS sample buffer (Thermo Scientific). Samples were heated at 95°C for 5 minutes. The entire sample volume was loaded on a 4–20% Mini-PROTEAN TGX Precast Protein Gel (Bio-Rad) in 1X Tris/glycine/SDS buffer and run at 40 mA constant until the dye front approached the end of the gel. The PAGE gel was removed from the electrophoresis rig and stained for one hour using GelCode Blue Stain Reagent (Thermo Scientific).

### Western blotting

For Western blotting, sample preparation and SDS-PAGE were performed as described above. Immobilon PVDF membrane (Millipore Sigma) was prepared by activation in 100% methanol for 1 minute, a 2-minute rinse in DI water, then a 10-minute incubation in Towbin transfer buffer (25 mM Tris, 192 mM glycine, 20% (v/v) methanol). The blotting sandwich was assembled and placed in the Trans-Blot Turbo Transfer System (Bio-Rad) for 30 minutes at 1.0 A maximum, 25V constant. The blot with transferred protein was removed and blocked at room temperature for 30 minutes with EveryBlot Blocking Buffer (Bio-Rad). The blot was removed and incubated with α-SERCA primary antibody (sc-271669, Santa Cruz Biotechnology) at a 1:500 dilution in 6 mL total volume of 1:1 TBS-T (20 mM Tris, 150 mM NaCl, 0.1% Tween 20 (w/v, Sigma-Aldrich)): EveryBlot Blocking Buffer overnight at 4°C. Primary antibody solution was removed, washed twice in 10 minute increments with TBS-T, and a final rinse in TBS. IRDye 800CW Goat anti-Mouse IgG secondary antibody (Licor) was diluted 1:15,000 in a TBS-T:Block solution and was incubated for 1 hour at room temperature on the blot. Following the previously described wash steps, the blot was shielded from light and dried at room temperature, then imaged using the Odyssey CLx imager (LICORbio).

### Electron microscopy

To examine venom composition and vesicle morphology, purified venom from 50 female wasps was prepared and imaged using electron microscopy techniques. For scanning electron microscopy (SEM), venom samples were filtered, fixed in 5% glutaraldehyde in 2X PBS, and imaged on a Helios NanoLab 650 Dual Beam FEG SEM (FEI/Thermo Fisher, Hillsboro, OR). For transmission electron microscopy (TEM), venom samples were filtered, and either fixed (whole venom, fractions F2-F5) in 5% glutaraldehyde in 2X PBS, or left unfixed (fraction F5). A drop of sample was placed on a glow-discharged grid, which was then briefly rinsed in a bead of water and stained with uranyl acetate. Excess stain was wicked off, and grids were imaged on a Titan G2 ChemiSTEM TEM (FEI/Thermo Fisher, Hillsboro, OR).

### Nanoparticle tracking analysis data collection

Venom samples were subjected to sucrose gradient fractionation, and five fractions were collected to isolate the venom vesicles. The collected fractions were then dialyzed against a 1X PBS solution to remove sucrose. Dialysis was performed using Slide-A-Lyzer MINI Dialysis Devices, 10K MWCO (Thermo Scientific), with two 1-hour room temperature exchanges in 1L PBS and an overnight exchange at 4°C in 1L PBS. Immediately prior to performing nanoparticle tracking analysis (NTA), dialyzed fractions were filtered using a 0.22 µm PES syringe filter (SFPES004022N, Membrane Solutions) and diluted 5-fold with PBS. The hydrodynamic diameters of vesicles were measured using a NanoSight LM10, configured with a temperature controlled LM14 sample viewing unit equipped with a 532 nm laser and a high sensitivity sCMOS camera (Malvern Panalytical). The temperature of the sample was held constant at 22°C, and analysis was performed under a constant flow rate of 10 μL/min to ensure representative sampling. NanoSight 3.1 software was used to collect five 60 second videos of each sample with the camera level set to 14. Using a detection threshold of 2, the mean hydrodynamic diameter and standard deviation of the vesicles in each sample were obtained from the analysis of approximately 10,000–55,000 individual vesicles, depending on the vesicle concentration in the collected fraction.

### Tunable resistive pulse sensing

For the assessment of venom vesicle characteristics, TRPS was performed using the Exoid (Izon). Venom was purified from 75 *G. hookeri* females and dialyzed in PBS in 10K MWCO Slide-A-Lyzer (Thermo Scientific) dialysis cups with two 2-hour buffer exchanges at room temperature, and a final exchange overnight at 4°C in fresh PBS. Following dialysis, venom samples were filtered with a 0.22 µm PES filter (SFPES004022N, Membrane Solutions). Samples were analyzed using an NP100 Nanopore (A97068, Izon) or NP250 Nanopore (A88596, Izon). Solutions were prepared according to manufacturer’s instructions with the following exception: for TPRS analysis of detergent treated venom, the SDS concentration of the measurement electrolyte was adjusted to equal the SDS concentration of the sample.

For size and concentration measurements of whole and fractionated venom, the NP100 Nanopore was calibrated with CPC 100 calibration particles and set to a stretch of 45 mm. For measurements of detergent treated venom, samples were measured using an NP250 nanopore at a 47 mm stretch. Calibration was performed utilizing CPC200 nanoparticles. Measurements were taken at three pressures: P1: 1000 Pa, P2: 800 Pa, and P3:1400 Pa, while a constant Nanopore stretch and constant 1100mV voltage were maintained throughout the measurement session. A minimum of 500 particles were counted at each pressure; the particle measurement rate never exceeded 880 particles/ minute, and the root mean square (RMS) noise never exceeded 12 pA to ensure measurement accuracy.

For zeta potential measurements, the NP100 Nanopore was calibrated with CPC 100 calibration particles and set to a stretch of 45 mm. Calibration analysis was performed at variable voltages and applied pressures (V1P1: 766 mV, 100 Pa; V1P0: 766 mV, 0 Pa; V2P0: 613 mV, 0 Pa; V3P0: 460 mV, 0 Pa). Sample measurements were taken at a constant Nanopore stretch of 45mm and constant 766 mV voltage, without any applied pressure (0 Pa). The average particle measurement rate was 29.3 particles/ minute.

Data were exported as comma-separated values (CSV) files using the Exoid Control Suite software (version 1.4). See [60] for a step-by-step protocol for TRPS measurements.

### *GCaMP3* calcium assays

To measure relative intracellular calcium abundance in hemocytes, the genetically encoded *UAS-GCaMP3* calcium sensor was crossed with the *Cg-GAL4* driver to express *GCaMP3* in plasmatocytes of the progeny. A total of 5 *Cg:GCaMP3* third-instar larvae were dissected in a single well of Tekdon (Tekdon, Inc) microscopy slides in 30 µL of PBS for each sample. Following a ten-minute incubation for cell adherence, the liquid component of each well was removed and 30 µL of venom fraction, heat denatured venom (heated at 90°C for 5 minutes), sonicated venom (water bath sonicated at full power for 15 minutes), or PBS control were applied to the hemocytes and incubated for 1 hour in the dark. This volume was then removed following incubation, and cells were fixed with 4% paraformaldehyde (w/v, Fisher Scientific) for 15 minutes. Fixative was removed and samples were washed thrice for 1 minute using PBS. Samples were mounted using VectaShield (VectorLabs) and imaged on a Keyence BZ-X810 Series microscope (Keyence Corporation) utilizing the GFP filter cube (Keyence, OP-87763), 20X objective (BZ-PA20; 20X/0.75, WD 0.6) and 40X objective (Keyence, BZPA40; 40X/0.95, WD 0.25-0.17).

### Sample preparation for MS analysis

35 µL of each sucrose gradient fraction in SDS sample buffer (2% SDS,10% glycerol, 50 mM Tris, 0.5X PBS, trace bromophenol blue) were separated onto a NuPAGE 10% Bis-Tris Protein gel (Cat. # NP0315BOX, Invitrogen) at 200V constant for 10min. The gel was stained using a Colloidal Blue Staining Kit (Cat. #LC6025, Invitrogen) following the manufacturer’s instruction. Each gel lane was excised and digested overnight at 37°C with Pierce Trypsin Protease, MS Grade (Cat. #90058, Thermo Scientific) as per manufacturer’s instruction. Digests were reconstituted in 0.1%FA in 5:95 ACN: ddH2O at ~0.1 µg/µL.

### nLC-ESI-MS2 analysis and database searches

Peptide digests (8 µL each) were injected onto a 1260 Infinity nHPLC stack (Agilent Technologies), and separated using a 100 µm inner diameter x 12.5 cm pulled tip C-18 column (JupiterC-18 300 Å, 5 microns, Phenomenex). This system runs in-line with a Thermo Orbitrap Velos Pro-hybrid mass spectrometer, equipped with a nano-electrospray source (Thermo Fisher Scientific), and all data were collected in CID mode. The nHPLC was configured with binary mobile phases that included solvent A (0.1% FA in ddH2O), and solvent B (0.1% FA in 15%ddH2O/ 85% ACN), programmed as follows; 10min @ 5%B (2µL/ min, load), 90min @ 5% -40%B (linear: 0.5nL/ min, analyze), 5min @ 70%B (2µL/ min, wash), 10min @ 0%B (2µL/min, equilibrate). Following each parent ion scan (300 -1200m/z @ 60k resolution), fragmentation data (MS2) was collected on the topmost intense 15 ions. For data-dependent scans, charge state screening and dynamic exclusion were enabled with a repeat count of 2, repeat duration of 30s, and exclusion duration of 90s.

The XCalibur RAW files were collected in profile mode, centroided and converted to MzXML using ReAdW v. 3.5.1. The data were searched using G1 protein sequences and SEQUEST, which was set for two maximum missed cleavages, a precursor mass window of20ppm, trypsin digestion, variable modification C @ 57.0293, and M @ 15.9949.

### Peptide filtering, grouping, and quantification

Peptides identified by mass spectrometry were mapped back onto the *G. hookeri* transcriptome (Sequence Read Archive: SRR805628) by SEQUEST (Thermo Fisher Scientific) search. SEQUEST results were filtered using Scaffold (Protein Sciences, Portland Oregon). Scaffold filters and groups all peptides to generate and retain only high confidence IDs while also generating normalized spectral counts (N-SC’s) across all samples for the purpose of relative quantification. The filter cut-off values were set with minimum peptide length of >5 AA’s, with no MH + 1charge states, with peptide probabilities of >80% C.I., and an FDR < 1.0%. Scaffold incorporates the two most common methods for statistical validation of large proteome datasets, the false discovery rate (FDR) and protein probability. Relative quantification across experiments was then performed via spectral counting, and when relevant, spectral count abundances were then normalized between samples. Mass spectrometry data are available via the Open Science Framework project F4jhv (https://doi.org/10.17605/OSF.IO/F4JHV).

### Bioinformatics analysis of identified venom proteins

Fraction membership was determined according to the following criteria: First, a protein must be identified by at least 2 unique peptides to count as present in a fraction. Second, the protein must have been identified in the whole venom control sample, or have a read coverage of at least 10 total peptides. Third, the total spectra count must be higher within the fraction than the average total spectra count across the fractions. These criteria allowed us to focus on proteins that have a high likelihood of presence in a given fraction and decreasing noise from any potential carryover between fractions.

To determine protein identities, we used the KofamKOALA webserver (https://www.genome.jp/tools/kofamkoala/; accessed 04–2025) to map our positively identified venom proteins onto established KEGG Orthology (KO) groups [36]. The KO assignments were additionally used for functional annotation using the KEGG Mapper Reconstruct tool (https://www.kegg.jp/kegg/mapper/reconstruct.html; accessed 04–2025) to identify protein “modules”, groups of proteins which form complete signaling or metabolic pathways [37,61]. We used FlyBase (release FB2025_02) to find information on *D. melanogaster* phenotypes and the integrated BLAST tool to map *G. hookeri* proteins onto *D. melanogaster* homologs [62].

### Statistical analysis

Statistical analyses were performed in R (RRID: SCR_001905) [63], and data were plotted using the ggplot2 (RRID: SCR_014601) and ggpubr (RRID: SCR_021139) packages [64,65].

Mixture modeling of diameter and zeta potential data from TRPS was performed using the mixtools package (v2.0.0.1) [66]. Recombined fractions were analyzed to reflect whole venom properties. Each model distribution is characterized by μ (population mean), σ (standard deviation) and λ (weight, or proportion of the total data points attributed to the distribution). We used a λ threshold of 10 (i.e., 10% of the vesicles in the population) to accept a distribution, and the resulting distributions were plotted by the plot_mix_comps command from plotGMM (v0.2.2) [67]. For fractions where diameter or zeta potential were monomodal, the summarySE function from Rmisc (v1.5.1) [68] was used to calculate the mean and standard deviation, which were overlaid on each histogram to summarize the spread of the data.

For the vesicle detergent sensitivity experiment, measured particle diameters were binned into 5nm intervals, and the generalized additive model was used to calculate a regression curve from the diameter vs concentration scatterplot in ggpubr. Linear regression lines were calculated in ggpubr for diameter vs zeta potential scatterplots. Pearson’s correlation between diameter and zeta potential was additionally calculated in ggpubr.

The molecular weight and pI of venom proteins identified by mass spectrometry were compared by one-way ANOVA followed by Tukey’s HSD *post hoc* testing for pairwise comparisons using multcomp (RRID: SCR_018255) [69]. The Venn diagram describing protein fraction membership was created using ggVennDiagram (v1.5.4) [70].

*Cg:GCaMP3* cell fluorescence intensity was measured FIJI [58] using the corrected total cell fluorescence protocol [14]. *Cg:GCaMP3* fluorescence was compared between control, fraction F5, and perturbation treatments on F5 using the Kruskal-Wallis test and Dunn’s *post hoc* test from the rstatix package (RRID: SCR_021240) [71].

## Supporting information

S1 Table Mass spectrometry results, showing all identified venom proteins with fraction membership. For positively identified proteins, the normalized total spectra counts are provided.
(XLSX)

S2 Table Small EV biomarker proteins identified in venom mass spectrometry. Membership in vesicle-containing fractions is indicated.
(XLSX)

S1 Movie Supporting information for Fig 6. Successive confocal micrograph Z-series images of Exo-Venom treated *hop[Tum]* hemocyte primary cultures taken at 63X magnification with a slice thickness of 330 nm. Intensity values have been quantized using a 16-color LUT, as in Fig 6C. Warmer colors indicate higher intensity values. Scale bar is 15 µm.
(AVI)

S1 Raw Images Raw images for Western blots and gels presented in the manuscript. (PDF)

S1 Fig Supporting information for Fig 1. Whole mount brightfield micrograph of the dissected venom apparatus shown in Fig 1A-C. Image is a single section taken at 40x magnification. Scale bar is 50 µm.
(TIF)

S2 Fig Supporting information for Venom purification methods. (A-B) Electron micrographs of unfixed, unfiltered venom showing vEV aggregation. Vesicles larger than 220 nm are not observed. Scale bars are 200 nm. (C) TRPS analysis of unfiltered and filtered whole venom. Aggregates represent a small portion of the particles analyzed in unfiltered venom. (D) SDS-PAGE analysis of unfiltered and filtered whole venom. Comparison of the banding patterns suggest that filtration has a minimal impact on venom contents, although an approximately 200 kDa band is selectively lost following filtration. We can conclude that filtration allows us to remove aggregated material without selectively losing or depleting vEVs.
(TIF)

S3 Fig Supporting information for Fig 3. (A) Box plot of protein molecular weight by fraction with individual data points overlaid. Letters indicate confidence groups assigned by Tukey’s HSD post hoc test. (B) Box plot of protein isoelectric point by fraction with individual data points overlaid. Letters indicate confidence groups assigned by Tukey’s HSD post hoc test.
(TIF)

S4 Fig Supporting information for Fig 4. Transmission electron micrograph of unfixed F5 vesicle displaying the unusual morphology. Scale bar is 100 nm.
(TIF)

S5 Fig Supporting information for Fig 6. Additional representative images of *hop[Tum]* cells incubated with ExoSparkler labeled venom (Exo-Venom; A-B) and ExoSparkler-PBS control (Exo-PBS; C-D). Confocal micrographs (A, C) shown with paired brightfield images (B, D). Scale bars are 20 µm.
(TIF)

## References

[1] Saunders TE, Ward DF. Variation in the diversity and richness of parasitoid wasps based on sampling effort. PeerJ. 2018;6:e4642. doi: 10.7717/peerj.4642 29632746 PMC5889912

[2] Asgari S, Theopold U, Wellby C, Schmidt O. A protein with protective properties against the cellular defense reactions in insects. Proc Natl Acad Sci U S A. 1998;95(7):3690–5. doi: 10.1073/pnas.95.7.3690 9520427 PMC19897

[3] Beckage NE. Modulation of immune responses to parasitoids by polydnaviruses. Parasitology. 1998;116 Suppl:S57-64. doi: 10.1017/s0031182000084948 9695110

[4] Moreau SJM, Asgari S. Venom proteins from parasitoid wasps and their biological functions. Toxins (Basel). 2015;7(7):2385–412. doi: 10.3390/toxins7072385 26131769 PMC4516919

[5] Rizki RM, Rizki TM. Parasitoid virus-like particles destroy *Drosophila cellular* immunity. Proc Natl Acad Sci U S A. 1990;87(21):8388–92. doi: 10.1073/pnas.87.21.8388 2122461 PMC54961

[6] Strand MR. Microplitis demolitor polydnavirus infects and expresses in specific morphotypes of pseudoplusia includens haemocytes. Journal of General Virology. 1994;75(11):3007–20. doi: 10.1099/0022-1317-75-11-3007 7964610

[7] Labrosse C, Eslin P, Doury G, Drezen JM, Poirié M. Haemocyte changes in D. Melanogaster in response to long gland components of the parasitoid wasp Leptopilina boulardi: A Rho-GAP protein as an important factor. J Insect Physiol. 2005;51(2):161–70. doi: 10.1016/j.jinsphys.2004.10.004 15749101

[8] Huang J, Chen J, Fang G, Pang L, Zhou S, Zhou Y, et al. Two novel venom proteins underlie divergent parasitic strategies between a generalist and a specialist parasite. Nat Commun. 2021;12(1):234. doi: 10.1038/s41467-020-20332-8 33431897 PMC7801585

[9] Ramroop JR, Heavner ME, Razzak ZH, Govind S. A parasitoid wasp of Drosophila employs preemptive and reactive strategies to deplete its host’s blood cells. PLoS Pathog. 2021;17(5):e1009615. doi: 10.1371/journal.ppat.1009615 34048506 PMC8191917

[10] Martinson EO, Wheeler D, Wright J, Siebert AL, Werren JH. Nasonia vitripennis venom causes targeted gene expression changes in its fly host. Mol Ecol. 2014;23(23):5918–30. doi: 10.1111/mec.12967 25319487 PMC5130349

[11] Song Y, Gu F, Zhou W, Li P, Wu F, Sheng S. Parasitoid wasps can manipulate host trehalase to the benefit of their offspring. Insects. 2022;13(9):833. doi: 10.3390/insects13090833 36135534 PMC9500599

[12] Kryukova NA, Yanshole LV, Zelentsova EA, Tsentalovich YP, Chertkova EA, Alekseev AA, et al. The venom of *Habrobracon hebetor* induces alterations in host metabolism. J Exp Biol. 2024;227(17):jeb247694. doi: 10.1242/jeb.247694 39253831

[13] Dong Z, Lu Y, Fang G, Zhang Q, Sheng Y, Pang L, et al. A rare, evolutionarily conserved venom protein benefits endoparasitism across parasitoids. Cell Genom. 2025;5(8):100920. doi: 10.1016/j.xgen.2025.100920 40541180 PMC12366661

[14] Mortimer NT, Goecks J, Kacsoh BZ, Mobley JA, Bowersock GJ, Taylor J, et al. Parasitoid wasp venom SERCA regulates *Drosophila calcium* levels and inhibits cellular immunity. Proc Natl Acad Sci U S A. 2013;110(23):9427–32. doi: 10.1073/pnas.1222351110 23690612 PMC3677475

[15] Kim-Jo C, Gatti J-L, Poirié M. *Drosophila cellular* immunity against parasitoid wasps: A complex and time-dependent process. Front Physiol. 2019;10:603. doi: 10.3389/fphys.2019.00603 31156469 PMC6529592

[16] Mortimer NT, Schlenke TA. Multifaceted defenses against parasitoid wasps in diptera. Annu Rev Genet. 2025;59(1):369–94. doi: 10.1146/annurev-genet-032425-100252 40825352 PMC12805944

[17] Hultmark D, Andó I. Hematopoietic plasticity mapped in Drosophila and other insects. Elife. 2022;11:e78906. doi: 10.7554/eLife.78906 35920811 PMC9348853

[18] Russo J, Dupas S, Frey F, Carton Y, Brehelin M. Insect immunity: early events in the encapsulation process of parasitoid (*Leptopilina boulardi*) eggs in resistant and susceptible strains of *Drosophila*. Parasitology. 1996;112 (Pt 1):135–42. doi: 10.1017/s0031182000065173 8587797

[19] Nappi AJ, Christensen BM. Melanogenesis and associated cytotoxic reactions: Applications to insect innate immunity. Insect Biochem Mol Biol. 2005;35(5):443–59. doi: 10.1016/j.ibmb.2005.01.014 15804578

[20] Mortimer NT, Kacsoh BZ, Keebaugh ES, Schlenke TA. Mgat1-dependent N-glycosylation of membrane components primes *Drosophila melanogaster* blood cells for the cellular encapsulation response. PLoS Pathog. 2012;8(7):e1002819. doi: 10.1371/journal.ppat.1002819 22829770 PMC3400557

[21] Alvarado G, Holland SR, DePerez-Rasmussen J, Jarvis BA, Telander T, Wagner N, et al. Bioinformatic analysis suggests potential mechanisms underlying parasitoid venom evolution and function. Genomics. 2020;112(2):1096–104. doi: 10.1016/j.ygeno.2019.06.022 31247332

[22] Altshuler I, Vaillant JJ, Xu S, Cristescu ME. The evolutionary history of sarco(endo)plasmic calcium ATPase (SERCA). PLoS One. 2012;7(12):e52617. doi: 10.1371/journal.pone.0052617 23285113 PMC3527596

[23] Heavner ME, Ramroop J, Gueguen G, Ramrattan G, Dolios G, Scarpati M, et al. Novel organelles with elements of bacterial and eukaryotic secretion systems weaponize parasites of *Drosophila*. Curr Biol. 2017;27(18):2869-2877.e6. doi: 10.1016/j.cub.2017.08.019 28889977 PMC5659752

[24] de Souza W, Barrias ES. Membrane-bound extracellular vesicles secreted by parasitic protozoa: Cellular structures involved in the communication between cells. Parasitol Res. 2020;119(7):2005–23. doi: 10.1007/s00436-020-06691-7 32394001

[25] Cruz-Bustos T, Feix AS, Hummel K, Schlosser S, Razzazi-Fazeli E, Joachim A. The proteomic landscape of *Toxoplasma gondii* extracellular vesicles across diverse host cell types. Front Cell Infect Microbiol. 2025;15:1565684. doi: 10.3389/fcimb.2025.1565684 40171158 PMC11958994

[26] Gueguen G, Rajwani R, Paddibhatla I, Morales J, Govind S. VLPs of *Leptopilina boulardi* share biogenesis and overall stellate morphology with VLPs of the heterotoma clade. Virus Res. 2011;160(1–2):159–65. doi: 10.1016/j.virusres.2011.06.005 21704090 PMC3905611

[27] Wan B, Goguet E, Ravallec M, Pierre O, Lemauf S, Volkoff A-N, et al. Venom atypical extracellular vesicles as interspecies vehicles of virulence factors involved in host specificity: The case of a Drosophila parasitoid wasp. Front Immunol. 2019;10:1688. doi: 10.3389/fimmu.2019.01688 31379874 PMC6653201

[28] Morales J, Chiu H, Oo T, Plaza R, Hoskins S, Govind S. Biogenesis, structure, and immune-suppressive effects of virus-like particles of a Drosophila parasitoid, *Leptopilina victoriae*. J Insect Physiol. 2005;51(2):181–95. doi: 10.1016/j.jinsphys.2004.11.002 15749103

[29] Chiu H, Morales J, Govind S. Identification and immuno-electron microscopy localization of p40, a protein component of immunosuppressive virus-like particles from *Leptopilina heterotoma*, a virulent parasitoid wasp of Drosophila. J Gen Virol. 2006;87(Pt 2):461–70. doi: 10.1099/vir.0.81474-0 16432035 PMC2705942

[30] Colinet D, Schmitz A, Depoix D, Crochard D, Poirié M. Convergent use of RhoGAP toxins by eukaryotic parasites and bacterial pathogens. PLoS Pathog. 2007;3(12):e203. doi: 10.1371/journal.ppat.0030203 18166080 PMC2156102

[31] Ferrarese R, Morales J, Fimiarz D, Webb BA, Govind S. A supracellular system of actin-lined canals controls biogenesis and release of virulence factors in parasitoid venom glands. J Exp Biol. 2009;212(Pt 14):2261–8. doi: 10.1242/jeb.025718 19561216 PMC2702457

[32] Welsh JA, Goberdhan DCI, O’Driscoll L, Buzas EI, Blenkiron C, Bussolati B, et al. Minimal information for studies of extracellular vesicles (MISEV2023): From basic to advanced approaches. J Extracell Vesicles. 2024;13(2):e12404. doi: 10.1002/jev2.12404 38326288 PMC10850029

[33] Hofmann G. ISCOtables a handbook of data for biological and physical scientists. 7th ed. Lincoln, Nebraska: Instrumentation Specialties Company; 1977.

[34] Brennan K, Martin K, FitzGerald SP, O’Sullivan J, Wu Y, Blanco A, et al. A comparison of methods for the isolation and separation of extracellular vesicles from protein and lipid particles in human serum. Sci Rep. 2020;10(1):1039. doi: 10.1038/s41598-020-57497-7 31974468 PMC6978318

[35] Rai A, Huynh K, Cross J, Poh QH, Fang H, Claridge B, et al. Multi-omics identify hallmark protein and lipid features of small extracellular vesicles circulating in human plasma. Nat Cell Biol. 2025;27(12):2167–85. doi: 10.1038/s41556-025-01795-7 41315767 PMC12717007

[36] Aramaki T, Blanc-Mathieu R, Endo H, Ohkubo K, Kanehisa M, Goto S, et al. KofamKOALA: KEGG Ortholog assignment based on profile HMM and adaptive score threshold. Bioinformatics. 2020;36(7):2251–2. doi: 10.1093/bioinformatics/btz859 31742321 PMC7141845

[37] Kanehisa M, Sato Y, Kawashima M. KEGG mapping tools for uncovering hidden features in biological data. Protein Sci. 2022;31(1):47–53. doi: 10.1002/pro.4172 34423492 PMC8740838

[38] Kanehisa M, Furumichi M, Tanabe M, Sato Y, Morishima K. KEGG: New perspectives on genomes, pathways, diseases and drugs. Nucleic Acids Res. 2017;45(D1):D353–61. doi: 10.1093/nar/gkw1092 27899662 PMC5210567

[39] Judge A, Dodd MS. Metabolism. Essays Biochem. 2020;64:607–47. doi: 10.1042/EBC20190041 PMC7545035 32830223

[40] Shimomura T, Seino R, Umezaki K, Shimoda A, Ezoe T, Ishiyama M, et al. New lipophilic fluorescent dyes for labeling extracellular vesicles: Characterization and monitoring of cellular uptake. Bioconjug Chem. 2021;32(4):680–4. doi: 10.1021/acs.bioconjchem.1c00068 33719402

[41] Hanratty WP, Dearolf CR. The *Drosophila Tumorous-lethal* hematopoietic oncogene is a dominant mutation in the hopscotch locus. Mol Gen Genet. 1993;238(1–2):33–7. doi: 10.1007/BF00279527 8479437

[42] Kurucz E, Váczi B, Márkus R, Laurinyecz B, Vilmos P, Zsámboki J, et al. Definition of *Drosophila* hemocyte subsets by cell-type specific antigens. Acta Biol Hung. 2007;58 Suppl:95–111. doi: 10.1556/ABiol.58.2007.Suppl.8 18297797

[43] Tattikota SG, Cho B, Liu Y, Hu Y, Barrera V, Steinbaugh MJ, et al. A single-cell survey of *Drosophila* blood. Elife. 2020;9:e54818. doi: 10.7554/eLife.54818 32396065 PMC7237219

[44] Heavner ME, Gueguen G, Rajwani R, Pagan PE, Small C, Govind S. Partial venom gland transcriptome of a *Drosophila* parasitoid wasp, *Leptopilina heterotoma*, reveals novel and shared bioactive profiles with stinging Hymenoptera. Gene. 2013;526(2):195–204. doi: 10.1016/j.gene.2013.04.080 23688557 PMC3905606

[45] Feix AS, Tabaie EZ, Singh AN, Wittenberg NJ, Wilson EH, Joachim A. An in-depth exploration of the multifaceted roles of EVs in the context of pathogenic single-cell microorganisms. Microbiol Mol Biol Rev. 2024;88(3):e0003724. doi: 10.1128/mmbr.00037-24 38869292 PMC11426017

[46] Dekel E, Yaffe D, Rosenhek-Goldian I, Ben-Nissan G, Ofir-Birin Y, Morandi MI, et al. 20S proteasomes secreted by the malaria parasite promote its growth. Nat Commun. 2021;12(1):1172. doi: 10.1038/s41467-021-21344-8 33608523 PMC7895969

[47] da Silva Lira Filho A, Fajardo EF, Chang KP, Clément P, Olivier M. Leishmania Exosomes/Extracellular Vesicles Containing GP63 Are Essential for Enhance Cutaneous Leishmaniasis Development Upon Co-Inoculation of Leishmania amazonensis and Its Exosomes. Front Cell Infect Microbiol. 2022;11:709258. doi: 10.3389/fcimb.2021.709258 35186777 PMC8851419

[48] Marcilla A, Trelis M, Cortés A, Sotillo J, Cantalapiedra F, Minguez MT, et al. Extracellular vesicles from parasitic helminths contain specific excretory/secretory proteins and are internalized in intestinal host cells. PLoS One. 2012;7(9):e45974. doi: 10.1371/journal.pone.0045974 23029346 PMC3454434

[49] Sánchez-López CM, González-Arce A, Ramírez-Toledo V, Bernal D, Marcilla A. Unraveling new players in helminth pathology: Extracellular vesicles from Fasciola hepatica and *Dicrocoelium dendriticum* exert different effects on hepatic stellate cells and hepatocytes. Int J Parasitol. 2024;54(12):617–34. doi: 10.1016/j.ijpara.2024.06.002 38925265

[50] Anderson W, Kozak D, Coleman VA, Jämting ÅK, Trau M. A comparative study of submicron particle sizing platforms: Accuracy, precision and resolution analysis of polydisperse particle size distributions. J Colloid Interface Sci. 2013;405:322–30. doi: 10.1016/j.jcis.2013.02.030 23759321

[51] Bachurski D, Schuldner M, Nguyen P-H, Malz A, Reiners KS, Grenzi PC, et al. Extracellular vesicle measurements with nanoparticle tracking analysis - An accuracy and repeatability comparison between NanoSight NS300 and ZetaView. J Extracell Vesicles. 2019;8(1):1596016. doi: 10.1080/20013078.2019.1596016 30988894 PMC6450530

[52] Vogel R, Savage J, Muzard J, Camera GD, Vella G, Law A, et al. Measuring particle concentration of multimodal synthetic reference materials and extracellular vesicles with orthogonal techniques: Who is up to the challenge?. J Extracell Vesicles. 2021;10:e12052. doi: 10.1002/jev2.12052 PMC7804049 33473263

[53] Maas SLN, Broekman MLD, de Vrij J. Tunable resistive pulse sensing for the characterization of extracellular vesicles. Methods Mol Biol. 2017;1545:21–33. doi: 10.1007/978-1-4939-6728-5_2 27943204

[54] Midekessa G, Godakumara K, Ord J, Viil J, Lättekivi F, Dissanayake K, et al. Zeta potential of extracellular vesicles: Toward understanding the attributes that determine colloidal stability. ACS Omega. 2020;5(27):16701–10. doi: 10.1021/acsomega.0c01582 32685837 PMC7364712

[55] Ghadami S, Dellinger K. The lipid composition of extracellular vesicles: Applications in diagnostics and therapeutic delivery. Front Mol Biosci. 2023;10:1198044. doi: 10.3389/fmolb.2023.1198044 37520326 PMC10381967

[56] Bretz N, Mortimer NT. Isolation of parasitoid wasp venom. 2025. doi: 10.17504/protocols.io.8epv5mprjl1b/v1

[57] Moller JV, Ning G, Maunsbach AB, Fujimoto K, Asai K, Juul B, et al. Probing of the membrane topology of sarcoplasmic reticulum Ca2+-ATPase with sequence-specific antibodies. Evidence for plasticity of the c-terminal domain. J Biol Chem. 1997;272(46):29015–32. doi: 10.1074/jbc.272.46.29015 9360975

[58] Schindelin J, Arganda-Carreras I, Frise E, Kaynig V, Longair M, Pietzsch T, et al. Fiji: An open-source platform for biological-image analysis. Nat Methods. 2012;9(7):676–82. doi: 10.1038/nmeth.2019 22743772 PMC3855844

[59] Sedgewick J. Using Photoshop with images created by a confocal system. Methods Mol Biol. 2014;1075:97–128. doi: 10.1007/978-1-60761-847-8_4 24052348

[60] Bretz N, Mortimer NT. Venom vesicle size and zeta potential analysis. 2025. https://protocols.io/q26g76j11vwz/v1

[61] Kanehisa M, Sato Y. KEGG Mapper for inferring cellular functions from protein sequences. Protein Sci. 2020;29(1):28–35. doi: 10.1002/pro.3711 31423653 PMC6933857

[62] Öztürk-Çolak A, Marygold SJ, Antonazzo G, Attrill H, Goutte-Gattat D, Jenkins VK, et al. FlyBase: Updates to the Drosophila genes and genomes database. Genetics. 2024;227(1):iyad211. doi: 10.1093/genetics/iyad211 38301657 PMC11075543

[63] R Core Team. R: A language and environment for statistical computing. Vienna, Austria: R Foundation for Statistical Computing; 2025.

[64] Wickham H. ggplot2: Elegant graphics for data analysis. New York: Springer; 2016. doi: 10.1007/978-0-387-98141-3

[65] Kassambara A. ggpubr: “ggplot2” based publication ready plots. 2025. https://CRAN.R-project.org/package=ggpubr

[66] Benaglia T, Chauveau D, Hunter DR, Young DS. Mixtools: An R package for analyzing mixture models. J Stat Softw. 2010;32:1–29. doi: 10.18637/jss.v032.i06

[67] Waggoner P, Chan F. plotGMM: Tools for Visualizing Gaussian Mixture Models. 2020. https://cran.r-project.org/web/packages/plotGMM/index.html

[68] Hope RM. Rmisc: Ryan miscellaneous. CRAN. 2022. https://cran.r-project.org/web/packages/Rmisc/index.html

[69] Hothorn T, Bretz F, Westfall P. Simultaneous inference in general parametric models. Biom J. 2008;50(3):346–63. doi: 10.1002/bimj.200810425 18481363

[70] Gao CH, Dusa A. ggVennDiagram: a “ggplot2” implement of venn diagram. 2025. https://cran.r-project.org/web/packages/ggVennDiagram/index.html

[71] Kassambara A. rstatix: Pipe-Friendly Framework for Basic Statistical Tests. 2025. https://cran.r-project.org/web/packages/rstatix/index.html

