# Supplementary material for: Venom vesicles from the parasitoid Ganaspis hookeri facilitate venom protein entry into host immune cells
Source: PLoS Pathog. 2026 Oct 16;22(10):e1014707. doi: 10.1371/journal.ppat.1014707 (PMC13693980; doi:10.1371/journal.ppat.1014707)

Raw image for Figure 3A. Stained with GelCode Blue Stain Reagent (Thermo Scientific), and imaged on an Odyssey CLx imager (Licor).

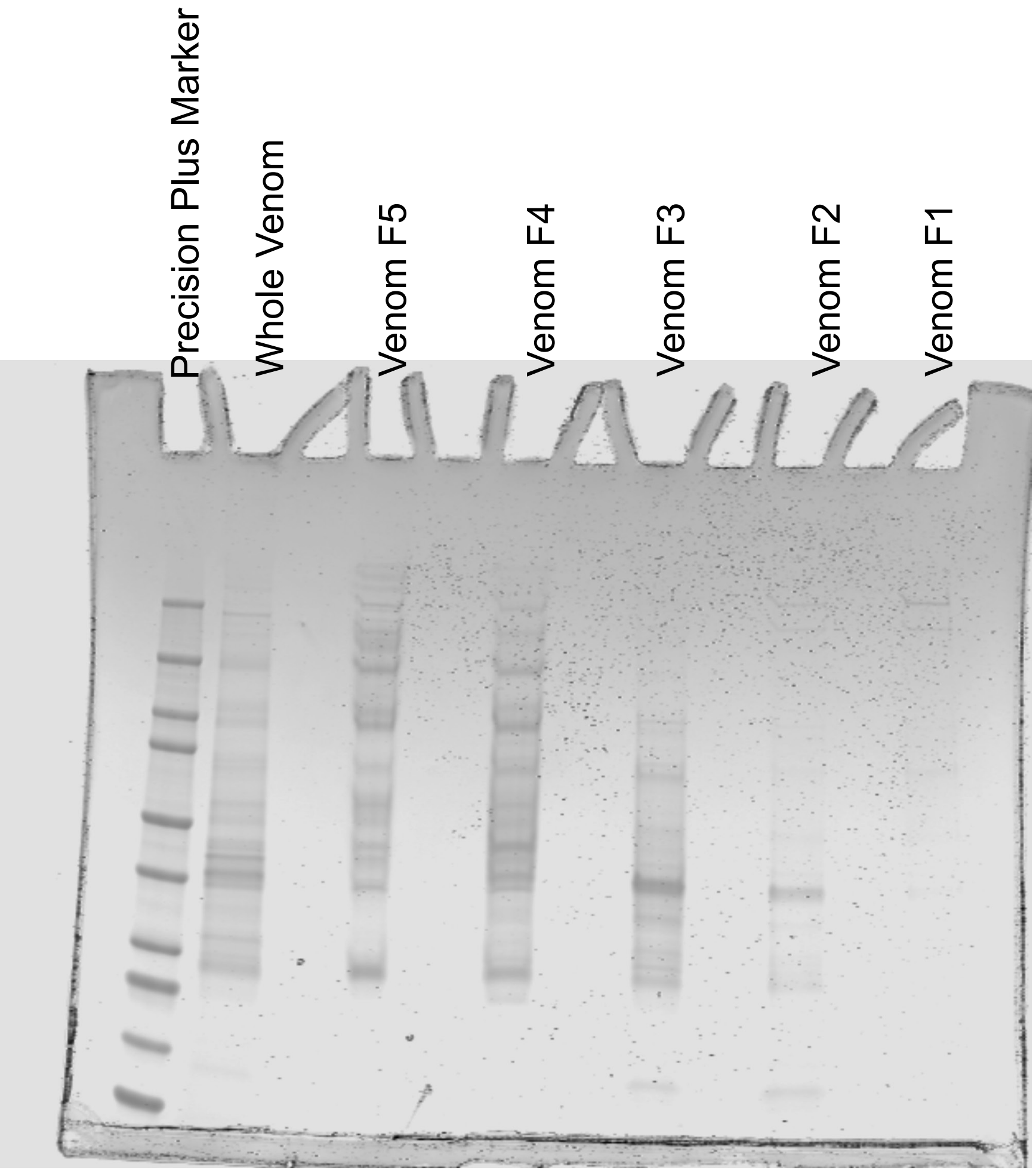

Raw image for Figure 3C. Venom fractions stained with anti-SERCA primary antibody, IRDye 800CW Goat anti-Mouse IgG secondary antibody and imaged on an Odyssey CLx imager (Licor).

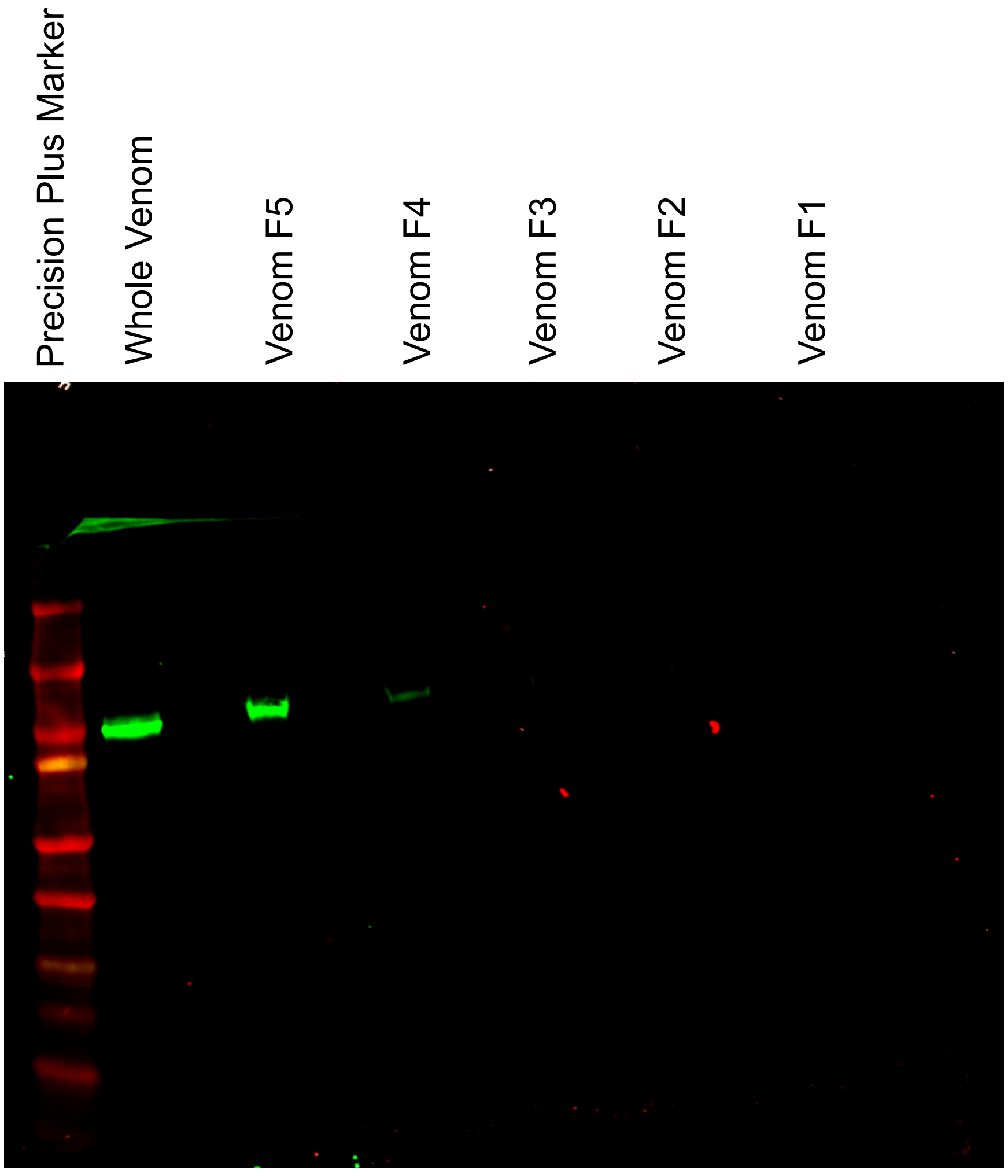

Raw image for Figure S2D. Stained with GelCode Blue Stain Reagent (Thermo Scientific), and imaged on an Odyssey CLx imager (Licor).

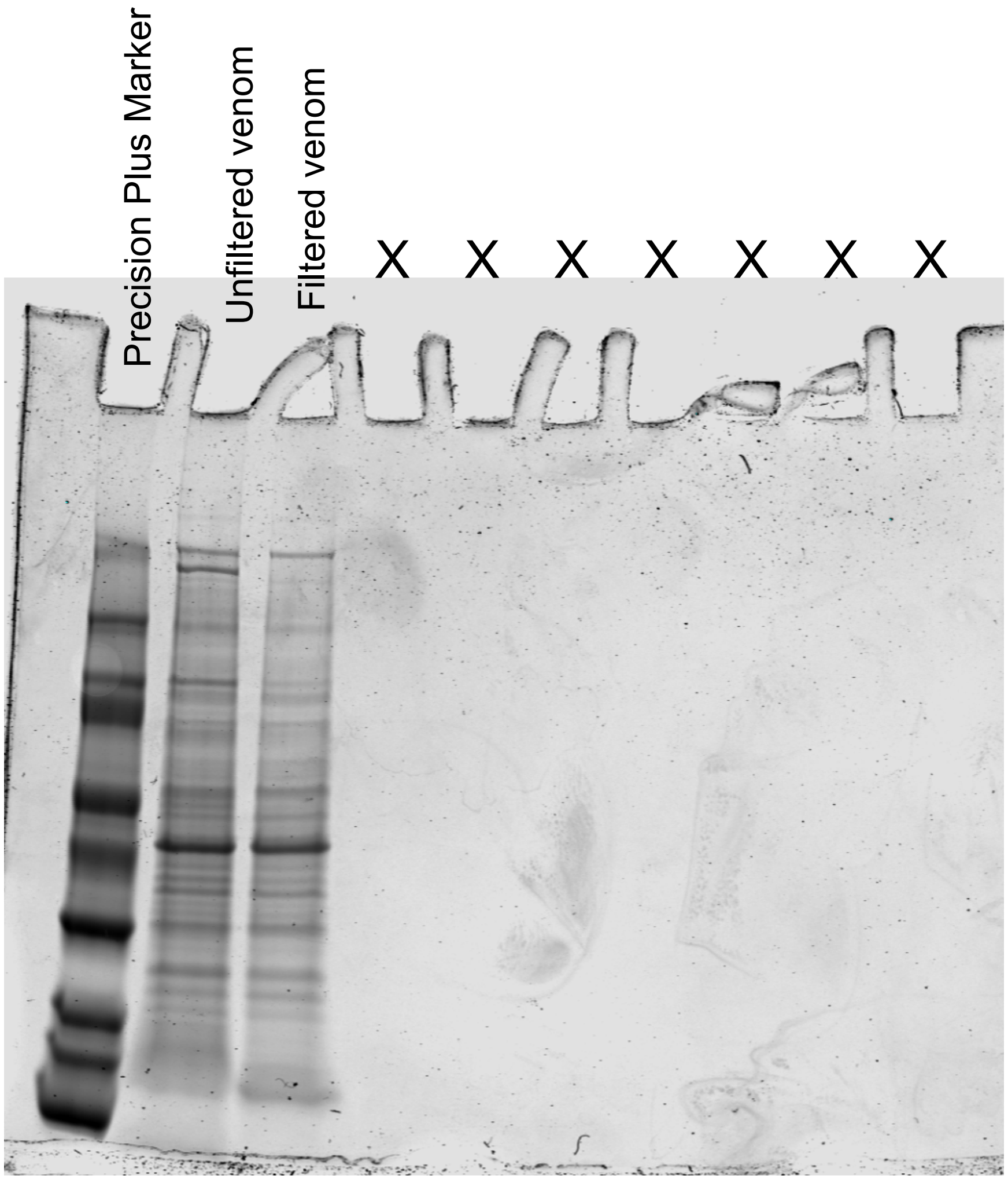

Supplement: Supplementary file 1 [file ppat.1014707.s004.pdf]
